# Nonlinear effects in Thomas precession due to the interplay of Lorentz contraction and Thomas–Wigner rotation

**DOI:** 10.1038/s41598-022-20942-w

**Published:** 2022-10-06

**Authors:** Antonio Di Lorenzo

**Affiliations:** Instituto de Física, Universidade Federal de Uberlândia, Av. João Naves de Ávila 2121, Uberlândia, Minas Gerais 38400-902 Brazil

**Keywords:** Theoretical physics, Physics

## Abstract

It is demonstrated that the 3-vector $\varvec{S}$ currently associated to the spin in an inertial frame does not contract, but rather dilates, in the direction of the velocity. The correct vector $\varvec{T}$ is individuated. The equation of motion for the two vectors is shown to contain two terms, a common linear rotation, identified with Thomas precession, and also a nonlinear rotation depending on the direction of the spin itself.

## Introduction

Often, particles carry with themselves properties, e.g. charge, mass, magnetic moment, electric dipole moment. While the motion of a particle $O'$ is described in a simpler form in an inertial reference frame, the evolution of physical quantities transported along $O'$ appears simpler when described in the comoving frame, even though it is an accelerated frame. Think, for instance, of a gyroscope attached to a car, which may accelerate, make a curve, brake, etc. Meanwhile, the car is being carried by Earth, which is spinning and rotating around the Sun, which in its turn is rotating around the center of the Galaxy. Even though the interior of the car is not an inertial reference frame, it is much easier to describe the motion of the gyroscope in such noninertial frame, after introducing noninertial forces that one attributes to gravitational fields, rather than describe its motion relative to the fixed stars. However, if from the car you look out of the window, all bodies not being transported along with you—trees, mountains, the Sun, the planets, the stars in the night sky—will appear to follow complicated, inexplicable motions, and there are no masses around compatible with the noninertial effects that you believed to be gravitational. If you want to simplify the description of the outside universe you should eventually switch to an inertial reference frame. The equations relating the change of the angular momentum of the gyroscope with the applied forces and torques should then be written back in an inertial frame, in which the movement of the origin of the gyroscope can be explained causally, i.e. all accelerations can be associated to other bodies, which are usually limited to nearby bodies due to the short range of all forces except gravity.

In a given reference frame, physical quantities are described by scalars, three-dimensional vectors, and $3\times 3$ matrices. The special theory of relativity, despite its name, allows to describe the equations of physics in an absolute form, by making use of abstract mathematical objects in Minkowski space, which encompasses space and time. Such objects—scalars, four-dimensional vectors, more generally tensors—describe absolute quantities. One can then deduce the physical quantities in any reference frame as representations of these absolute objects.

In our example, the angular momentum of the gyroscope, in the reference frame inside the car, is represented as a 3-vector ${\varvec{S}}'$, which is a representation $\{0,S'^1,S'^2,S'^3\}$ of the abstract 4-vector $\underline{S}$ that is being transported along the car. In another reference frame, the same 4-vector is represented differently, as $\{S^0,S^1,S^2,S^3\}$. Here, the first component is the time component.

## Results

**Figure 1:**
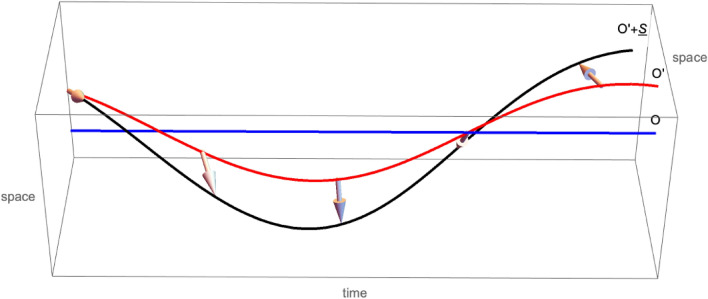
A vector field (arrows, sampled at discrete times) is being transported along an accelerated worldline $O'$ (red) as represented by an inertial observer *O* (blue). Its tip traces a line (black) close to $O'$. The vector field is normal to the worldline $O'$ in the Minkowski metric, hence it does not appear as orthogonal in our Euclidean space representation.

With reference to Fig. 1 we ask: given the 4-vector field $\underline{S}_t$ transported along a worldline $O'$ in Minkowski space, normally to the world line, so that it is represented as a purely spatial vector, a 3-vector, in the comoving frame ${\mathfrak {S}}'$, how is it represented, as a 3-vector, in an inertial frame ${\mathfrak {S}}$? what are the equations of motion for the new representation, supposing the equations in the comoving frame to be known? These questions are particularly relevant in atomic physics, since an electron orbiting an atom carries an intrinsic magnetic moment.

Our first result is that the relativity of simultaneity implies that the spin, as a 3-vector in the lab frame, is not the one which has appeared so far in the literature, consisting in the spatial part $\varvec{S}$ of the 4-vector representation $\{S^0,\varvec{S}\}$. Indeed, $\varvec{S}$ does not undergo Lorentz contraction in the direction of motion, as a properly defined 3-vector should. On the contrary, it dilates in the direction of motion.

The correct 3-vector to describe the spin in the lab frame, instead, is $\varvec{T}=\varvec{S}- S^0\varvec{v}/c^2$. Because the spin is normal to the world line of the particle, in an inertial frame, $S^0=\varvec{v}{\boldsymbol{\cdot }}\varvec{S}$, while in a general frame $S^0=(\varvec{v}-\boldsymbol{\Omega }\times \varvec{r}){\boldsymbol{\cdot }}\varvec{S}/[(1-\varvec{g}{\boldsymbol{\cdot }}\varvec{r})^2-(\boldsymbol{\Omega }\times \varvec{r})^2+(\boldsymbol{\Omega }\times \varvec{r}){\boldsymbol{\cdot }}\varvec{v}]$, with $\varvec{g}$ the equivalent gravitational field, $\boldsymbol{\Omega }$ the gravi-magnetic Coriolis field, and $\varvec{r}$ the position of the spin. The combinations $\theta =1-\varvec{g}{\boldsymbol{\cdot }}\varvec{r}$ and $\varvec{w}=\boldsymbol{\Omega }\times \varvec{r}$ are to be identified with the scalar and the vector potential of the gravitational field.

The previous literature uses $\varvec{S}$ as the spin, and it is common to indicate as $\varvec{V}$ the spatial components of a 4-vector $\vec {V}$, hence we are keeping the symbol $\varvec{S}$ for the conventional spin for ease of comparison. We shall also write formulas for both the spin definitions. We shall see later on that, irrespectively of the procedure used, the linear rotation, seen in an inertial reference frame, of the 3-vectors $\varvec{S}$ and $\varvec{T}$ is the same.

Our second result is that both vectors $\varvec{S}$ and $\varvec{T}$, in the lab frame, obey a differential equation which contains a linear rotation term and also a nonlinear term. The linear term, which we identify with Thomas precession, differs from the accepted value. We explain that the difference is due to the fact that the accepted value of Thomas precession does not describe the rotation of the spin in the lab frame, but rather is the rate of the Coriolis rotation of an auxiliary frame which is purely rotating relative to the lab.

### Detailed results

Let us provide more details. According to a naive approach, which takes into account only the relativistic transformation of the electromagnetic field, the spin-orbit term in the Hamiltonian for the electron should be about twice as large as the one observed. A full relativistic treatment, however, reproduces the correct spin-orbit coupling, as shown by Thomas^1,2^. The additional precession, due exclusively to relativistic effects for an accelerated frame, has been calculated in various ways, and there are some discrepancies in the literature. The common value accepted for the precession is^2,3^

1 $$\begin{aligned} \boldsymbol{\omega }^\mathrm {Th} {\mathop {=}\limits ^{?}} -\frac{\gamma -1}{v^2} {\varvec{v}}\boldsymbol{\times }{\varvec{a}}=-\frac{\gamma ^2}{(\gamma +1)c^2} {\varvec{v}}\boldsymbol{\times }{\varvec{a}} , \end{aligned}$$

with $\varvec{v}$ and $\varvec{a}$ the Newtonian 3-velocity and 3-acceleration, *c* the speed of light, and $\gamma =(1-v^2/c^2)^{-1/2}$ the Lorentz factor. There are several other expressions for Thomas precession in the literature, some even with opposite signs, due mainly to some different interpretation or downright misinterpretation^4,5^. While the work of Malykin^4^ reports exhaustively and critically the values that have been proposed, it ends up agreeing on the value as proposed by Thomas^2^. In the following, we shall show that the accepted factor is incorrect, in the sense that Eq. (1) does not describe the precession of the spin as seen in an inertial frame, e.g. the lab frame: because of Lorentz contraction, the spin of an accelerated particle makes a complicated motion, which is not a simple precession. Instead, we prove that Eq. (1) describes rather the rotation, relative to the lab frame, of the axes of a rotating frame (whose origin coincides with that of the lab frame). The relative rotation rate is thus a Coriolis rotation. This fact has been occasionally pointed out in the literature^6–8^.

We show that, in the lab frame, the motion of the direction of the spin

2 $$\begin{aligned} \frac{d}{dt}{\hat{\varvec{S}}}_t=[\boldsymbol{\omega }^\mathrm {Th}(t)+\boldsymbol{\omega }^\mathrm {nl}(t,{\hat{\varvec{S}}}_t)]\times {\hat{\varvec{S}}}_t \end{aligned}$$

contains a linear kinematic rotation,

3 $$\begin{aligned} \boldsymbol{\omega }^\mathrm {Th} = -\frac{\gamma ^2}{2c^2} {\varvec{v}}\boldsymbol{\times }{\varvec{a}} , \end{aligned}$$

but also an additional nonlinear precession, which, for a planar motion, is

4 $$\begin{aligned} \boldsymbol{\omega }^\mathrm {nl} = \frac{\gamma ^2}{c^2} \left[ \varvec{v}{\boldsymbol{\cdot }}\varvec{a}\, {\hat{\varvec{v}}}{\boldsymbol{\cdot }}{\hat{\varvec{S}}}\,{\hat{\varvec{n}}}{\boldsymbol{\cdot }}{\hat{\varvec{S}}} +\frac{1}{2}\kappa v^3 \left( {\hat{\varvec{n}}}{\boldsymbol{\cdot }}{\hat{\varvec{S}}}\,{\hat{\varvec{n}}}{\boldsymbol{\cdot }}{\hat{\varvec{S}}}-{\hat{\varvec{v}}}{\boldsymbol{\cdot }}{\hat{\varvec{S}}}\,{\hat{\varvec{v}}}{\boldsymbol{\cdot }}{\hat{\varvec{S}}}\right) \right] {\hat{\varvec{b}}}. \end{aligned}$$

Here $\kappa$ is the curvature, ${\hat{\varvec{v}}}$ the tangent, ${\hat{\varvec{n}}}$ the normal, and ${\hat{\varvec{b}}}={\hat{\varvec{v}}}\boldsymbol{\times }{\hat{\varvec{n}}}$ the binormal of the trajectory described in the lab frame. The nonlinearity is a consequence of splitting the motion of the spin in an equation for its norm and an equation for its direction, as detailed in the “Methods” section. The overall equation for the spin vector, however, is linear, and it is but the Bargmann–Michel–Telegdi equation^9^ represented in the lab frame. The presence of a nonlinearity was found out by Stepanov^8^ in a particular case, and also by Kholmetskii and Yarman^10^.

Furthermore, we shall prove that for a uniform circular motion, for which the linear precession rate (3) is constant, the average contribution of the nonlinear term (4), when added to the linear term yields Eq. (1). This apparently fortuitous occurrence may be at the origin of the unquestioned standing of Eq. (1).

Our Eq. (3) has a simple geometrical interpretation for planar motions: the angle of linear precession during a time *T* is equal to the area swiped by the celerity $\gamma \varvec{v}$ divided by $c^2$. The corresponding lowest order limit, with the factor $\gamma =1$, was mentioned by Borel^11,12^ already in 1913. Thus, apparently, Borel had derived at least an approximate expression for Thomas precession, using merely geometrical considerations.

In solving the problem of Thomas precession, we used a coordinate-free approach to the problem, which allows us to identify a skew-symmetric tensor $\underline{\underline{{\mathfrak {B}}}}$, the generator of Fermi–Walker transport, depending only on the intrinsic geometry of the worldline of the accelerated particle, which describes a kinematic rotation in Minkowski space. The coordinate-free approach is extremely valuable, in that it is robust against error, and in that it provides a manifestly invariant picture, in the spirit of Minkowski^13^ and of the reference book by Misner, Thorne, and Wheeler^14^. Often we read that the equations of physics must be covariant. Actually, covariance implies already the choice of a representation, where the physical quantities are represented by lists with upper or lower indexes, which transform contravariantly or covariantly. However vectors and tensors are invariant objects, only their representations are contravariant or covariant.

In the lab frame, the motion of the spin is dictated by

5a $$\begin{aligned} \partial _t \varvec{S}_t&= \frac{q}{m} \biggl \{\frac{g_\mathrm {L}}{2\gamma _t}\left[ \varvec{S}_t\boldsymbol{\times }\varvec{B}_t+\varvec{S}_t{\boldsymbol{\cdot }}\varvec{v}_t \, \varvec{E}_t\right] -\left( \frac{g_\mathrm {L}}{2}-1\right) \varvec{S}_t{\boldsymbol{\cdot }}\varvec{G}_t\, \varvec{v}_t \biggr \}\ , \end{aligned}$$

5b $$\begin{aligned} \partial _t \varvec{T}_t&= \frac{q}{m}\biggl \{\frac{g_\mathrm {L}}{2\gamma _t}\left[ \varvec{T}_t\boldsymbol{\times }\varvec{B}_t-\varvec{T}_t{\boldsymbol{\cdot }}\varvec{E}_t\,\varvec{v}_t\right] +\left( \frac{g_\mathrm {L}}{2}-1\right) \varvec{T}_t{\boldsymbol{\cdot }}\varvec{v}_t\,\varvec{G}_t\biggr \}\ . \end{aligned}$$

Equation (5a) is the spatial part of the Bargmann–Michel–Telegdi equation represented in the lab frame. Here, the term not multiplied by $g_\mathrm {L}$, $(q/m)\varvec{S}_t{\boldsymbol{\cdot }}\varvec{G}_t\, \varvec{v}_t$, identifies the kinematic contribution due to the spin being accelerated; the other terms are due to the direct coupling with the electromagnetic field. The 3-vector $\varvec{G}=\gamma \left[ \varvec{E}-(\varvec{E}{\boldsymbol{\cdot }}\varvec{v}/ c^{2})\varvec{v}+\varvec{v}\boldsymbol{\times }\varvec{B}\right]$ is the simultaneity-corrected Lorentz force per unit charge.

### Case studies

Consider a particle making a planar motion, as described in an inertial frame by the trajectory ${\varvec{r}}_t$. Let ${\hat{\varvec{v}}}_t, {\hat{\varvec{n}}}_t, {\hat{\varvec{b}}}$ the Frenet–Serret apparatus for the curve ${\varvec{r}}_t$. As shown in the “Methods” section, the simplest description of the motion of the spin is that in the comoving frame whose axes are Fermi–Walker (FW) transported^15,16^. The FW axes can be calculated, in the lab frame representation, for a general two-dimensional motion, as

6a $$\begin{aligned} {\vec {e}\,}'_{\!(1)}&=\begin{pmatrix}\gamma _t\cos {(\psi _t)} v_t\\ \gamma _t\cos (\psi _t){\hat{\varvec{v}}}_t -\sin {(\psi _t)}{\hat{\varvec{n}}}_t\end{pmatrix}, \end{aligned}$$

6b $$\begin{aligned} {\vec {e}\,}'_{\!(2)}&= \begin{pmatrix}\gamma _t\sin {(\psi _t)} v_t\\ \gamma _t\sin {(\psi _t)}{\hat{\varvec{v}}}_t+\cos {(\psi _t)}{\hat{\varvec{n}}}_t\end{pmatrix}, \end{aligned}$$

6c $$\begin{aligned} {\vec {e}\,}'_{\!(3)}&=\begin{pmatrix}0\\ {\hat{\varvec{b}}}\end{pmatrix}, \end{aligned}$$

where $\psi _t$ is the angle $\psi _t=\int ^t \gamma _t \kappa _t v_t dt$, with $\kappa _t$ the curvature of ${\varvec{r}}_t$. The Thomas–Wigner rotation ${\mathcal {R}}_t$ can be calculated explicitly in this case,

7 $$\begin{aligned} {\mathcal {R}}_t= \begin{pmatrix} \cos (\psi _t-\alpha _t)&{}-\sin (\psi _t-\alpha _t)&{}0\\ \sin (\psi _t-\alpha _t)&{}\cos (\psi _t-\alpha _t)&{}0\\ 0&{}0&{}1 \end{pmatrix} , \end{aligned}$$

where $\alpha _t=\int ^t \kappa _t v_t dt$ is the Darboux rotation angle, i.e. the angle that the tangent ${\hat{\varvec{v}}}_t$ makes with the *X* axis.

#### Uniform circular motion

Let us consider the simple case of a particle, carrying an intrinsic angular momentum $\underline{S}$, in uniform circular motion, so that $\gamma =const$ and $\kappa =const$. The Thomas–Wigner rotation ${\mathcal {R}}_t$ in this case is

8 $$\begin{aligned} {\mathcal {R}}_t= \begin{pmatrix} \cos [(\gamma -1)\omega t]&{}-\sin [(\gamma -1)\omega t]&{}0\\ \sin [(\gamma -1)\omega t]&{}\cos [(\gamma -1)\omega t]&{}0\\ 0&{}0&{}1 \end{pmatrix}, \end{aligned}$$

while the FW axes are

9a $$\begin{aligned} {\vec {e}\,}'_{\!(1)}&=\begin{pmatrix}\gamma \cos (\gamma \omega t)\, v\\ \gamma \cos (\gamma \omega t){\hat{\varvec{v}}}_t -\sin {(\gamma \omega t)}{\hat{\varvec{n}}}_t\end{pmatrix}, \end{aligned}$$

9b $$\begin{aligned} {\vec {e}\,}'_{\!(2)}&= \begin{pmatrix}\gamma \sin (\gamma \omega t)\, v\\ \gamma \sin {(\gamma \omega t)}{\hat{\varvec{v}}}_t+\cos {(\gamma \omega t)}{\hat{\varvec{n}}}_t\end{pmatrix}, \end{aligned}$$

9c $$\begin{aligned} {\vec {e}\,}'_{\!(3)}&=\begin{pmatrix}0\\ {\hat{\varvec{b}}}\end{pmatrix}. \end{aligned}$$

Thus, for a motion with frequency $\omega$, a vector $\underline{S}=X'_{t'} \underline{e}_{(1)}+Y'_{t'} \underline{e}_{(2)}+Z'_{t'} \underline{e}_{(3)}$ with components $X'_{t'},Y'_{t'},Z'_{t'}$ with respect to the FW basis yields in the lab frame a vector moving according to:

10a $$\begin{aligned} \varvec{S}&= \gamma \left[ X'_{t'}\cos (\gamma \omega t)+Y'_{t'}\sin (\gamma \omega t)\right] {\hat{\varvec{v}}}_t -\left[ X'_{t'}\sin (\gamma \omega t)-Y'_{t'}\cos (\gamma \omega t)\right] {\hat{\varvec{n}}}_t + Z'_{t'} {\hat{\varvec{b}}} \ , \end{aligned}$$

10b $$\begin{aligned} \varvec{T}&= \gamma ^{-1}\left[ X'_{t'}\cos (\gamma \omega t)+Y'_{t'}\sin (\gamma \omega t)\right] {\hat{\varvec{v}}}_t -\left[ X'_{t'}\sin (\gamma \omega t)-Y'_{t'}\cos (\gamma \omega t)\right] {\hat{\varvec{n}}}_t + Z'_{t'} {\hat{\varvec{b}}} , \end{aligned}$$

where $$t'=\tau (t)=t/\gamma$$.

We fixed the origin of time so that

11 $$\begin{aligned} {\hat{\varvec{v}}}_t=\cos (\omega t) {\hat{\boldsymbol{\imath }}}+\sin (\omega t) {\hat{\boldsymbol{\jmath }}},\qquad {\hat{\varvec{n}}}_t=-\sin (\omega t) {\hat{\boldsymbol{\imath }}} +\cos (\omega t) {\hat{\boldsymbol{\jmath }}}. \end{aligned}$$

#### Spin with no magnetic moment

We shall consider $\lambda =0$, so that the spin of the particle does not couple to the field causing the motion, and thus only the kinematic term contributes to its time evolution. Then, the terms $X', Y', Z'$ in Eqs. (10) are constant.

**Figure 2:**
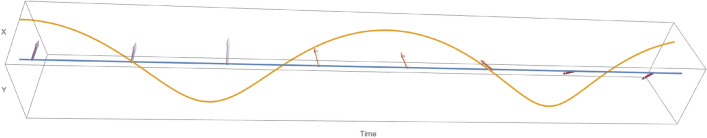
The parametric plot of the in-plane components $\varvec{T}_\parallel (t)$ of the spin ${\varvec{T}}$ carried along a point making a uniform circular motion, as seen from an inertial observer at the center of the motion. We used $v=3c/5$.

**Figure 3:**
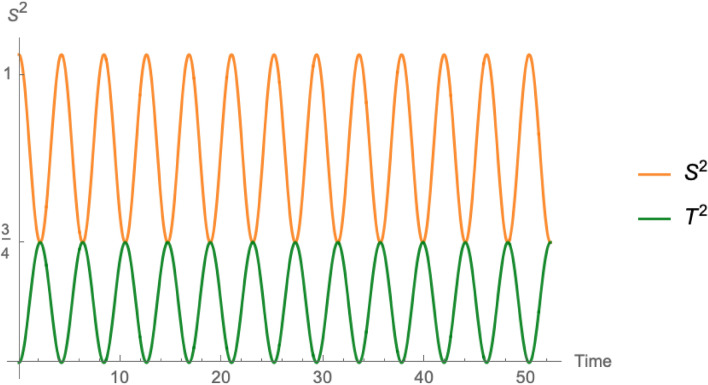
The variation in time of the total spin squared $\varvec{S}^{2}$ and $\varvec{T}^{2}$ divided by $\hbar ^{2}$. For the sake of comparison, we considered the values $X'=1/\sqrt{2}, Y'=0, Z'=1$, as in^14^ (pp. 175–176). We put $v=3c/5$ to make the effect of Lorentz dilation/contraction evident. As discussed in the text, in an atom the speed of the orbiting electrons is too small compared to *c* to allow to detect the effect.

A parametric plot of this solution is provided in Fig. 2. The squared norm $T^2$ of the simultaneity-corrected spin oscillates between the maximum, intrinsic value of the spin, $X'^2+Y'^2+Z'^2$ and the minimum $[X'^2+Y'^2]/\gamma ^2+Z'^2$, obtained when the in-plane component is parallel to the velocity; correspondingly, the squared norm $S^2$ of the conventional spin reaches a minimum $X'^2+Y'^2+Z'^2$ and a maximum, $\gamma ^2[X'^2+Y'^2]+Z'^2$, as illustrated in Fig. 3.

For the external electrons orbiting a nucleus the velocity is approximately $v\simeq Z_\mathrm {eff}(n)\alpha c/n$, where $\alpha \simeq 1/137$ is the fine structure constant, *n* the principal orbital number, and $Z_\mathrm {eff}(n)$ the effective nuclear number seen in the orbital *n* because of the screening. The contraction effect is very small, of order $5{\boldsymbol{\times }}10^{-5}$. For inner shell electrons of a heavy nucleus, where the effective charge of the nucleus is increased up to *Z*, the effect is a little larger, reaching 1 part in 1000.

The solution ${\varvec{S}}$ is provided in^ 14^ (pp. 175–176), as an exercise. The authors of^14^ arrive to the conclusion that the precession rate is $(\gamma -1)\omega =\gamma ^{2} v^{2}\omega /[c^2(\gamma +1)]$, by splitting the oscillation in a steady precession and a term that, it is argued, averages to zero.

Indeed, according to the decomposition outlined in the “Methods” section, the linear part of the angular velocity is $\boldsymbol{\omega }^{\mathrm {Th}}=\omega ^{\mathrm {Th}}{\hat{\varvec{b}}}$, with $\omega ^{\mathrm {Th}}=-(\gamma ^2-1)\omega /2$, while the nonlinear part for the conventional ($+$ sign) and the simultaneity-corrected (− sign) spin is $\boldsymbol{\omega }^{\mathrm {nl},\pm }=\omega ^{\mathrm {nl},\pm } {\hat{\varvec{b}}}$ with

12 $$\begin{aligned} {\omega }^{\mathrm {nl},\pm }&= \pm \frac{\gamma ^2}{2} [\varvec{a}_t{\boldsymbol{\cdot }}{\hat{\varvec{P}}}^\pm _t\, {\hat{\varvec{P}}}^\pm _t\boldsymbol{\times }\varvec{v}_t +\varvec{v}_t {\boldsymbol{\cdot }}{\hat{\varvec{P}}}^\pm _t\, {\hat{\varvec{P}}}^\pm _t\boldsymbol{\times }\varvec{a}_t]{\boldsymbol{\cdot }}{\hat{\varvec{b}}} \nonumber \\&= \pm \frac{\gamma ^2-1}{2} \frac{(\gamma ^{\pm 2}+1)\cos ^2(\gamma \omega t-\phi )-1}{(\gamma ^{\pm 2}-1)\cos ^2(\gamma \omega t-\phi )+1} \omega , \end{aligned}$$

with $\tan (\phi )=Y'/X'$ and ${\hat{\varvec{P}}}^\pm$ the unit vector of the in-plane component of the spin. Thus $\boldsymbol{\omega }^\mathrm {nl}$ oscillates with a fundamental frequency $2\gamma \omega$. The average value over a period is

13 $$\begin{aligned} {\overline{\omega }}^{\mathrm {nl},\pm }=\frac{(\gamma -1)^2}{2}\omega \ , \end{aligned}$$

thus it contributes a higher order correction, with respect to the linear term, in the non-relativistic limit. Remarkably, the average contribution is the same for both the conventional and the simultaneity-corrected spin.

When this average correction is added to the linear part, we get, for a lucky coincidence, the accepted value of Thomas precession,

14 $$\begin{aligned} \overline{\boldsymbol{\omega }}_\mathrm {tot}=-\frac{1}{2}\left[ \gamma ^2-1-(\gamma -1)^2\right] \omega {\hat{\varvec{b}}}=-(\gamma -1)\omega {\hat{\varvec{b}}} . \end{aligned}$$

Thus, we can say that the accepted result for Thomas precession holds, as an average, for uniform circular orbits, when adding the constant linear and the average nonlinear contribution to the precession.

#### Spin with magnetic moment in a cyclotron

In the lab frame, let $\varvec{B}=B{\hat{\varvec{b}}}$ a uniform magnetic field and let the electric field $\varvec{E}=\varvec{0}$. Let the lab frame the frame in which the particle is making a uniform circular motion (and not a helix) with cyclotron frequency $\omega = -q B/\gamma m$ (a positive charge will rotate clockwise as seen from the positive semi-axis of the magnetic field, hence the minus sign). Contrary to the previous case, we do not put $\lambda =0$, but rather $\lambda =g_\mathrm {L} q/2m$, so that the term due to the magnetic field now contributes as well to the time-evolution of the spin. This case is relevant for muon and electron experiments^17–19^.

In the comoving FW frame the electromagnetic field is

15 $$\begin{aligned} \varvec{B}'=\gamma \varvec{B} , \qquad \varvec{E}' = {\mathcal {R}}_{t'}(\gamma \varvec{v}_t\boldsymbol{\times }\varvec{B}) , \end{aligned}$$

with ${\mathcal {R}}_{t'}$ given in Eq. (8). Thus

16 $$\begin{aligned} \varvec{E}' = \gamma v B [ \sin (\gamma \omega t){\hat{\boldsymbol{\imath }}}-\cos (\gamma \omega t){\hat{\boldsymbol{\jmath }}}] . \end{aligned}$$

From the point of view of the particle, the force $q\varvec{E}'$ due to the electric field is compensated by the force $m\varvec{g}$ due to the local gravitational field^20^ $\varvec{g}=-\varvec{a}'_\mathrm {prop}$, where $\varvec{a}'_\mathrm {prop}$ is commonly called the proper acceleration (the particle however, does not feel an acceleration, since it is by definition at rest in its comoving frame: it feels a gravitational field, which has the dimension of an acceleration due to the equivalence of inertial and gravitational mass).

In the comoving FW frame, according to Eq. (43) derived in the “Methods” section, the spin precesses with angular velocity $g_\mathrm {L}\gamma ^2 \omega /2$,

17a $$\begin{aligned} X'_{t'}&= S'_\parallel \cos \left( \frac{g_\mathrm {L}}{2}\gamma ^2\omega t'+\phi \right) , \end{aligned}$$

17b $$\begin{aligned} Y'_{t'}&= S'_\parallel \sin \left( \frac{g_\mathrm {L}}{2}\gamma ^2\omega t'+\phi \right) , \end{aligned}$$

17c $$\begin{aligned} Z'_{t'}&= Z'_0 , \end{aligned}$$

with $S'_\parallel = \sqrt{X'^2+Y'^2}=\sqrt{S'^2-Z'^2}$ the constant in-plane component of the spin, and $\phi$ a constant depending on the initial conditions. Replacing these equations in Eq. (10), with $t=\gamma t'$, we obtain the time evolution in the lab frame for a magnetic moment in a cyclotron,

18a $$\begin{aligned} \varvec{S}&= S'_\parallel \left[ \gamma \cos (a \gamma \omega t+\phi ){\hat{\varvec{v}}}_t +\sin (a\gamma \omega t+\phi ){\hat{\varvec{n}}}_t\right] + Z' {\hat{\varvec{b}}} \ , \end{aligned}$$

18b $$\begin{aligned} \varvec{T}&= S'_\parallel \left[ \gamma ^{-1} \cos (a \gamma \omega t+\phi ){\hat{\varvec{v}}}_t +\sin (a\gamma \omega t+\phi ){\hat{\varvec{n}}}_t\right] + Z' {\hat{\varvec{b}}} \ , \end{aligned}$$

with $a=(g_\mathrm {L}-2)/2$ the *g*-factor anomaly. Thus, if the *g*-factor were exactly 2, i.e. $a=0$, the spin would stay rigidly fixed with respect to the Frenet–Serret apparatus formed by the tangent, the normal, and the binormal of the trajectory.

The linear precession rate is

19 $$\begin{aligned} \boldsymbol{\omega }^\mathrm {Th} = [1+\tfrac{1}{2} (\gamma ^2+1)a] \omega {\hat{\varvec{b}}}, \end{aligned}$$

while the nonlinear precession rate is

20 $$\begin{aligned} \boldsymbol{\omega }^{\mathrm {nl},\pm } = {\mp }\frac{1}{2}(\gamma ^2-1) a\omega \frac{({\hat{\varvec{v}}}_t{\boldsymbol{\cdot }}{\hat{\varvec{P}}}^\pm _t)^2-({\hat{\varvec{n}}}_t{\boldsymbol{\cdot }}{\hat{\varvec{P}}}^\pm _t)^2}{({\hat{\varvec{v}}}_t{\boldsymbol{\cdot }}{\hat{\varvec{P}}}^\pm _t)^2+({\hat{\varvec{n}}}_t{\boldsymbol{\cdot }}{\hat{\varvec{P}}}^\pm _t)^2} {\hat{\varvec{b}}}, \end{aligned}$$

where as in the previous case $\varvec{P}^\pm$ stands for the conventional or the simultaneity corrected spin, depending on the index being $+$ or −, respectively.

After replacing Eq. (18) into Eq. (20), we have

21 $$\begin{aligned} \boldsymbol{\omega }^{\mathrm {nl},\pm } = {\mp }\frac{1}{2}(\gamma ^2-1)a \omega \frac{(\gamma ^{\pm 2}+1)\cos ^2(a\gamma \omega t+\phi )-1}{(\gamma ^{\pm 2}-1)\cos ^2(a\gamma \omega t+\phi )+1} {\hat{\varvec{b}}} . \end{aligned}$$

A time-average over a period $T=\pi /a\gamma \omega$ yields

22 $$\begin{aligned} \overline{\boldsymbol{\omega }}^{\mathrm {nl}\pm } =-\frac{1}{2}a(\gamma -1)^2\omega {\hat{\varvec{b}}} , \end{aligned}$$

so that the overall average precession is

23 $$\begin{aligned} \overline{\boldsymbol{\omega }}_\mathrm {tot} = (1+a\gamma )\omega {\hat{\varvec{b}}}. \end{aligned}$$

As in the former case, this is the value obtained using the incorrect formula for Thomas precession.

The fluctuations are

24 $$\begin{aligned} \Delta \omega = \sqrt{\frac{\gamma }{2}}(\gamma -1)a\omega . \end{aligned}$$

#### Non-circular orbits

For a general orbit where the linear precession rate $(\gamma _t^2/2) \varvec{v}_t\boldsymbol{\times }\varvec{a}_t$ is not constant, adding an average contribution $\overline{\boldsymbol{\omega }}^\mathrm {nl}$ can not lead to the accepted formula $\boldsymbol{\omega }_\mathrm {tot}=-[\gamma ^2_t/(\gamma _t+1)]\varvec{v}_t\times \varvec{a}_t$. As an example, we take the relativistic orbits in a Coulomb potential, as discussed by Darwin and Sommerfeld^21,22^.

For simplicity, we will consider $\lambda =0$. After a few calculations, the Thomas precession term is found to be

25 $$\begin{aligned} \omega ^\mathrm {Th}=-\frac{h}{2\rho ^3}=-\frac{L e^2}{2m^2 c^2r^3} , \end{aligned}$$

where $\rho$ the distance from the center *r* in units of the electron radius, $R_0=e^2/mc^2$. while *h* is the angular momentum *L* in units of $mR_0c$, with *e* the charge of the electron, and *m* its mass.

The non-linear precession has a more involved expression, which we will provide for the simultaneity-corrected spin only:

26 $$\begin{aligned} \omega ^\mathrm {nl,-}=-\frac{u^2}{2}\frac{\frac{hu}{(\epsilon +u)^2}\cos ^2{\psi _u}-hu\sin ^2{\psi _u} + 2 \frac{\sqrt{Q^*_u}}{(\epsilon +u)^3} \sin {\psi _u}\cos {\psi _u}}{\frac{1}{(\epsilon +u)^2}\cos ^2{\psi _u}+\sin ^2{\psi _u}}, \end{aligned}$$

where $u=1/\rho$, $\epsilon$ is the total energy in units of the rest energy $mc^2$, while $Q^*_u=\epsilon ^2-1+2\epsilon u+(1-h^2)u^2$ is a quadratic form in *u*, and

27 $$\begin{aligned} \psi _u&= \frac{h}{h^2-1}\sqrt{Q^*_u}+\frac{h\epsilon }{(h^2-1)^{3/2}} \arctan \left[ \frac{\epsilon +(1-h^2)u}{\sqrt{(h^ 2-1)Q^*_u}}\right] \nonumber \\&\quad -\frac{1}{2} \arctan \left[ \frac{(\epsilon -1)(1+h^2u)+u}{(\epsilon -1)h\sqrt{Q^*_u}}\right] -\frac{1}{2}\arctan \left[ \frac{(\epsilon +1)(1+h^2u)+u}{(\epsilon +1)h\sqrt{Q^*_u}}\right] . \end{aligned}$$

## Discussion

In conclusion, the accepted value for the Thomas precession stems from confusing it with the rate of Thomas–Wigner rotation, which applies in a rotating frame possibly convenient but of no physical significance.

Instead, we wish to know the equations of motion of the spin either in the comoving frame the axes of which undergo FW transport, or in the lab frame. In the former frame, the equation takes its simplest form, Eq. (43), in the latter frame either Eq. (44a) or (44b) holds, depending if one is using the conventional definition of spin $\varvec{S}$ or the simultaneity-corrected one $\varvec{T}$.

The reason behind the complicated motion in the lab frame is that a Lorentz boost introduces a rotation: indeed, if a vector $\varvec{S}'$ stays constant in the rest frame of the particle, when going to the lab frame one has to apply a Thomas–Wigner rotation and a boost. The boost changes the component of $\varvec{S}$ along the velocity, thus the vector will undergo an additional rotation. This rotation is nonlinear, as it depends on the orientation of $\varvec{S}'$ relative to the velocity. In Section C of the Supplemental Materials we show in detail how the contribution of the Thomas–Wigner rotation and the contribution of Lorentz contraction get twined.

In a uniform circular motion, the effect of the Lorentz contraction averages out, because the Lorentz contraction factor is constant and because the spin, over time, points in all directions. This exceptional case, however, has been mistaken for the norm, since the uniform circular motion is the simplest curvilinear accelerated motion, and hence is the go-to case when treating Thomas precession.

While in the lab frame the spin does not undergo a simple precession, we have shown how to extract univocally from the equation of motion a linear rotation term, which we identify with the Thomas precession, and a nonlinear rotation term, due to the interplay of Lorentz contraction and Thomas–Wigner rotation.

In perspective, it is interesting to consider the expansion of the present results to the general relativistic case, along the lines of Ref. ^23^, where the Thomas precession combines with de Sitter and Lense–Thirring precession.

## Methods

### Relativity of simultaneity

Given the 4-vector field $\underline{S}_t$ along the trajectory of a particle $O'$, what is the spin, as a three-dimensional vector, that an observer *O* will assign? The conventional procedure is to consider the representation, in the frame of *O*, $\vec {S}=\{S^0,\varvec{S}\}$, and take the spatial part $\varvec{S}$ as the spin. (We have introduced the symbol $\vec {S}$ for the contravariant representation, in a given reference frame, of the abstract vector $\underline{S}$. We shall keep the concepts of a vector and of its representation distinct, as explained in the Supplemental Materials.) However, since *O* and $O'$ are in relative motion, the relativity of simultaneity applies, which results in a different 3-vector $\varvec{T}$ to be associated by the observer *O*.

**Figure 4:**
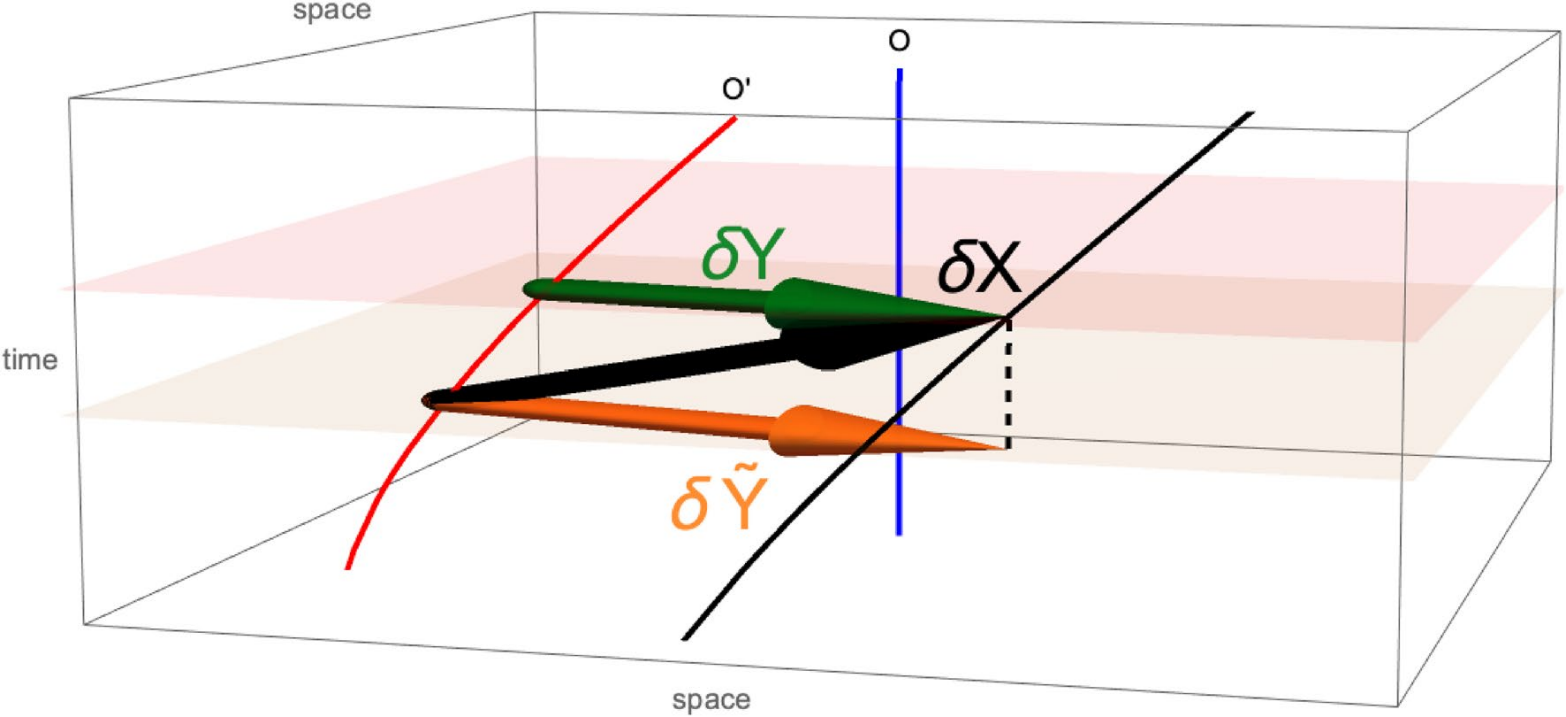
There are two procedures that an inertial observer can use to build a vector field normal to the worldline *O* from the vector field normal to $O'$ (black arrow): by projecting it on the simultaneity eigenplane passing through the origin of the vector (orange), or by building a vector joining the points at which the hyperplane of simultaneity crosses $O'$ and the line traced by the tip. We note that the time component gives a negative contribution to the squared length of a vector, thus the orange vector is larger than the black vector (by a Lorentz factor $\gamma$, to be precise). On the other hand, the shortening in space of the green vector more than compensates for its increase due to the suppression of the time component, yielding a contraction by a factor $\gamma$ instead.

To illustrate this point, we consider an infinitesimal vector in Minkowski space, $\underline{\delta X}_{t'}$, transported along $O'$. The tip of this vector points to $P'_{t'}=O'_{t'}+\underline{\delta X}_{t'}$ from the point $O'_{t'}$. The tip of the vector thus traces a curve $P'$ close to $O'$. For an inertial observer *O*, whose worldline is a straight line, the spatial vector to associate is $\underline{\delta Y}_t$, obtained by intersecting the two curves $O'$ and $P'=O'+\underline{\delta X}$ with a hyperplane of simultaneity $t=const$, then considering the infinitesimal vector from the intersection $O'_t$ to the intersection $P'_t$. By construction, in the inertial frame the representation of $\underline{\delta Y}_t$ is purely spatial $\vec {\delta Y}=\{0,\boldsymbol{\delta Y}_t\}$.

If $\underline{\delta X}$ is a rod of intrinsic length $\delta l=\sqrt{\underline{\delta X}{\boldsymbol{\cdot }}\underline{\delta X}}$ carried by the accelerated observer, the corresponding vector $\boldsymbol{\delta Y}$ describes a rod contracted by the Lorentz factor $|\boldsymbol{\delta Y}|=\delta l/\gamma$. If, instead, we applied the conventional procedure to a rod, rather than contracting, it would dilate in the direction of motion, $|\boldsymbol{\delta X}|=\gamma \delta l$, as illustrated in Fig. 4.

The procedure accounting for the relativity of simultaneity applies to any vector field transported normally to a worldline $O'$. We shall refer to it as the simultaneity-corrected procedure. Two other important vector fields normal to the worldline of a particle are its 4-acceleration and the Lorentz 4-force on it. In a frame where their representation is $\{A^0,\varvec{A}\}$ and $\{F^0,\varvec{F}\}$, the 3-vectors conventionally associated with these fields are $\varvec{A}=\gamma ^2 \varvec{a}+\frac{\gamma ^4}{c^2}\varvec{a}{\boldsymbol{\cdot }}\varvec{v}\,\varvec{v}$ and $\varvec{f}=\varvec{F}/\gamma =\varvec{E}+\varvec{v}\boldsymbol{\times }\varvec{B}$. We note how the definitions are mutually inconsistent, due to the extra $1/\gamma$ factor in the force $\varvec{f}$, which is introduced in order to keep the form of Newton’s second law $\varvec{f}=\frac{d}{dt} \varvec{p}$.

The simultaneity corrected 3-vectors are instead $\varvec{A}^\mathrm {sim}=\gamma ^2\varvec{a}$ and $\varvec{G}=\gamma \left[ \varvec{E}-\varvec{E}{\boldsymbol{\cdot }}\varvec{v}\,\frac{\varvec{v}}{c^2}+\varvec{v}\boldsymbol{\times }\varvec{B}\right]$, with $\varvec{E},\varvec{B}$ the electric and magnetic field. Newton’s second law becomes then: $q\varvec{G}=m\varvec{A}^\mathrm {sim}$.

In the case of spin, we opt for the simultaneity-corrected procedure because the intrinsic magnetic moment of the electron, in the Gilbert model, might be due to two equal and opposite magnetic monopoles separated by a small distance. (Precisely, if we take into account Dirac’s quantization and take the monopoles to be elementary, the separation explaining the observed magnetic dipole moment is the classical radius of the electron $\delta r=e^2/(mc^2)$.) Thus, the magnetic moment should behave as a rod.

If instead the magnetic moment is due to the rotation of a charge, as in the Ampère model, consider the following: a distributed charge is making a circular motion around its center of mass in a given frame. In a frame where the center of mass moves with speed $\varvec{v}$ normal to the plane of circular motion, the charges will make a helical trajectory, while their angular speed is reduced by a factor $\gamma$ due to the dilation of time, and the magnetic moment is reduced correspondingly.

The magnetic moment, therefore, decreases by a factor $1/\gamma$ in the direction of motion, irrespective of the underlying model.

A further argument in favour of using the simultaneity-corrected spin $\varvec{T}$ is the following. The magnetic dipole moment $\varvec{M}$ is obtained by the integration over space of the magnetic dipole density $\varvec{m}$. The latter is a 3-vector, which, together with the electric dipole density $\varvec{p}$, forms the skew-symmetric electromagnetic dipole tensor $$d=(\varvec{p},\varvec{m}):=\begin{pmatrix}0&{}\varvec{p}^\mathrm {tr}\\ \varvec{p}&{}\varvec{m}\cdot \varvec{{\mathcal {J}}}\end{pmatrix}$$. (We are representing *d* as a matrix, $d^\alpha _{\,\beta }$, i.e. a contravariant-covariant tensor, thus the skew-symmetry is not reflected in the components mixing time and space). The electric and magnetic dipole density 4-vectors are obtained by *d* and its dual $$*d=\begin{pmatrix}0&{}\varvec{m}^\mathrm {tr}\\ \varvec{m}&{}-\varvec{p}\cdot \varvec{{\mathcal {J}}}\end{pmatrix}$$ by contraction with the 4-velocity, $p^\alpha =d^\alpha _{\,\beta }U^\beta$, $m^\alpha =*d^\alpha _{\,\beta }U^\beta$, hence they are automatically normal to the 4-velocity. In the rest frame of the electron $\varvec{p}'=0$, as no intrinsic electric dipole of the electron has been observed so far. Therefore, in the lab frame, recalling that the Lorentz transformation from the lab frame to the electron rest frame includes the Thomas–Wigner rotation, $L_t=R_t B[\varvec{v}_t]$, with $B[\varvec{v}]$ a Lorentz boost, the magnetic dipole moment density is $\varvec{m}= {\mathcal {R}}_t^\mathrm {tr}\varvec{m}'-(\gamma _t-1){\hat{\varvec{v}}}_t\boldsymbol{\times }({\hat{\varvec{v}}}_t\boldsymbol{\times }{\mathcal {R}}_t^\mathrm {tr}\varvec{m}')$. The component of ${\mathcal {R}}_t^\mathrm {tr}\varvec{m}'$ parallel to the velocity is unaffected by the Lorentz contraction, ${\hat{\varvec{v}}}_t{\boldsymbol{\cdot }}\varvec{m}= {\hat{\varvec{v}}}_t{\boldsymbol{\cdot }}({\mathcal {R}}_t^\mathrm {tr}\varvec{m}')$, while the component perpendicular to the velocity is multiplied by a factor $\gamma$. Upon integration over the volume occupied by the electron, we get finally

28 $$\begin{aligned} \varvec{M}= & {} \int dV \varvec{m}= \int \frac{dV'}{\gamma _t}[{\mathcal {R}}_t^\mathrm {tr}\varvec{m}'+(\gamma _t-1){\hat{\varvec{v}}}_t\boldsymbol{\times }({\hat{\varvec{v}}}_t\boldsymbol{\times }{\mathcal {R}}_t^\mathrm {tr}\varvec{m}')]\nonumber \\= & {} \frac{{\mathcal {R}}_t^\mathrm {tr}\varvec{M}'}{\gamma _t}+\frac{\gamma _t-1}{\gamma _t}{\hat{\varvec{v}}}_t\boldsymbol{\times }({\hat{\varvec{v}}}_t\boldsymbol{\times }{\mathcal {R}}_t^\mathrm {tr}\varvec{M}'). \end{aligned}$$

Thus, the total dipole magnetic moment contracts by a factor $\gamma$ in the direction of the velocity. In the rest frame of the electron $\varvec{M}'=\lambda \varvec{S}'$, with $\lambda = g_\mathrm {L} e/(2m)$, $e=-|e|$ being the signed charge of the electron. In order for the same proportionality to apply in the lab frame, we must have $\varvec{M}=\lambda \varvec{T}$, i.e. the simultaneity-corrected spin must be used.

As a test of which definition is relevant, consider a beam of electrons at relativistic speed. It is possible to polarize the electrons longitudinally, so that their spin is parallel to their velocity and their magnetic moment is antiparallel to it. If the beam enters a region of magnetic field *B* parallel to the spin, a Zeeman splitting occurs, with the electrons in the upper level, along with a reduction of the initial speed due to the magnetic field gradient. If the spin, and hence the magnetic moment, were increased by a factor $\gamma$, then the deceleration of the electrons should be accordingly large, and also the frequency of the photons emitted in the relaxation to the lower Zeeman state should be large. For instance, in a 1GeV linear accelerator, electrons can reach a factor $\gamma \simeq 2\times 10^3$, making the effect, or the lack thereof, easily detectable.

We denoted the 3-dimensional vector field constructed according to the relativity of simultaneity as $\varvec{T}$,

29 $$\begin{aligned} \varvec{T} = \varvec{S}-S^0 \varvec{v} = \varvec{S}- \varvec{S}{\boldsymbol{\cdot }}\varvec{v}\, \varvec{v} \ . \end{aligned}$$

From here on, we shall use units where $c=1$. We also note that, in an inertial frame, the scalar product of the two definitions yields the constant squared norm of the spin

30 $$\begin{aligned} \varvec{S}{\boldsymbol{\cdot }}\varvec{T} = \underline{S}{\boldsymbol{\cdot }}\underline{S} = \frac{3}{4}\hbar ^2 . \end{aligned}$$

### Fermi–Walker transport

Fermi–Walker (FW) transport^15,16^ has a crucial role in the following. We illustrate it right away.

Consider a particle, having a velocity $\varvec{v}_{t_0}$, relative to an inertial frame ${\mathfrak {S}}$, at time $t_0$ in the inertial frame. Let us pick an inertial reference frame ${\mathfrak {S}}'_{t_0}$ where the particle is at rest at time $t_0$, and let $t'_0$ the proper time. At an infinitesimally later time $\delta t'$ in ${\mathfrak {S}}'_{t_0}$, the velocity of the particle changes by $\delta \varvec{v}' = \varvec{a}'_{t'_0} \delta t'$, with $\varvec{a}'_{t'_0}$ the proper acceleration. Now, there are infinitely many different inertial reference frames where the particle is at rest at time $t'_0+\delta t'$, which can be obtained by applying an infinitesimal Lorentz boost followed by an arbitrary rotation of the spatial axes. A Lorentz boost does not rotate the spatial axes normal to the velocity, and it rotates the time axis and the spatial axis parallel to the velocity in their common plane. It is therefore the simplest Lorentz transformation. (Lorentz boosts are the transformations often presented in introductory texts on special relativity as the Lorentz transformations *tout court*. Actually, they form a subset, which is not a subgroup, of Lorentz transformations: in special relativity, the 3D space is a hyperplane normal to the worldline of the origin of a reference frame. Two reference frames in relative motion will thus have different 3D spaces. These spaces, however, will share a common plane. Lorentz boosts are transformations between reference frames in which two of the spatial axes, usually the *Y* and *Z* axes, belong to the common plane and coincide between the two frames.)

Thus, let us consider, among these infinitely many frames, the one which is obtained by a pure infinitesimal boost with velocity $\boldsymbol{\delta v}'$. Let us repeat the procedure at another infinitesimal time $\delta t''$, etc. The sequence of infinitesimal boosts defines FW transport. Since the boosts do not form a subgroup, their product is a general Lorentz transformation, which can be written as the product of a 3D rotation and a boost.

**Figure 5:**
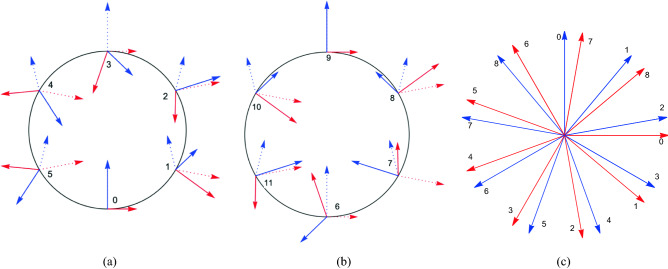
(**a**,**b**). For a particle in uniform circular motion, the *X* (red) and *Y* (blue) axes will make a complicated motion as seen from the lab frame. In the article, we show how to extract a linear rotation, which we identify with Thomas precession, and an additional nonlinear rotation. The solid lines represent the axes of a Fermi–Walker accelerated frame, the dotted lines the axes of a boosted frame, defined by pure Lorentz boosts applied to the lab frame. The length of the axes is constant in the accelerated frame, but varies in the lab frame due to Lorentz contraction. The speed is $v=4c/5$, thus $\gamma =5/3$. The axes were chosen to coincide with the lab axes at $t=0$, when the particle is at $\{0,-R\}$. The position of the axes is shown at times $t=n T/6$, with *T* the period in the lab frame, for (**a**) $n=0,1,\dots ,5$ and (**b**) $n=6,7,\dots , 11$. (**c**) In the frame defined by the dotted axes, the boosted frame, the solid axes appear to be purely rotating, with a frequency $\omega '=\gamma (\gamma -1)\omega$, hence a period $T'=9T/10$. Due to the time dilation, in the boosted frame the axes of the FW frame will appear as shown in the subfigure at times $t'=nT/10=nT'/9$.

In particular, if the spatial axes of the reference frame comoving with the particle are FW transported, they individuate a special accelerated reference frame, the FW frame, which is non-rotating. This means that, if we spin three gyroscopes attached to the particle, then accelerate the particle without applying any torque to the gyroscopes, the axes of these will be FW transported. While the gyroscopes do not rotate in the sense that a simple Lorentz boost is applied from an instantaneous comoving frame to the next, when viewing them from the initial reference frame ${\mathfrak {S}}$, they will make a complicated motion in which we shall individuate a linear precession term, the Thomas precession. It is erroneous, however, to state that the gyroscope will just appear as precessing in the lab frame. On one hand, Lorentz contraction will affect the gyroscopes, on the other hand, due to the relativity of simultaneity, the angles among them will appear to change in time in the lab frame, as shown in Fig. 5.

In any other accelerated frame following the same worldline, but with axes not being FW transported, an additional Coriolis rotation term appears, causing an apparent precession of the gyroscopes in the accelerated frame in absence of external torques. In this sense, the FW frame is minimally non-inertial. The Fermi–Walker (FW) reference frame, ${\mathfrak {S}}'_{t}$, thus has a special status among the frames comoving with an accelerated particle: no Coriolis forces appear in it. More details can be found in the Supplemental Materials.

The Lorentz transformation from the lab frame to the FW frame $L_\mathrm {FW}$ can be decomposed either as $R_\mathrm {TW}(t) B[\varvec{v}_t]$ or as $B[{\mathcal {R}}_\mathrm {TW}(t)\varvec{v}_t] R_\mathrm {TW}(t)$. The $4\times 4$ matrix $R_\mathrm {TW}(t)$ is a rotation in 3D space, the Thomas–Wigner rotation, its nontrivial part being the $3\times 3$ orthogonal matrix ${\mathcal {R}}_\mathrm {TW}(t)$. The $4\times 4$ matrix $B[\varvec{v}_t]$ is a Lorentz boost.

While the matrix $R_\mathrm {TW}(t)$ is the same in both decompositions, it represents two distinct physical rotations.

In the decomposition $L_\mathrm {FW}= R_\mathrm {TW}(t) B[\varvec{v}_t]$, the rotation is applied after the Lorentz boost, hence it represents the rotation of the axes of the FW frame relative to another frame, comoving with the particle, defined by the application of Lorentz boosts from the lab frame: the boosted rest frame, ${\mathfrak {S}}'^*_{t}$. The rate of rotation $\Omega '$ is obtained by differentiating in the proper time $t'$, $\frac{d}{dt'}{\mathcal {R}}_\mathrm {TW}(\tau ^{-1}(t'))= \Omega '(t'){\mathcal {R}}_\mathrm {TW}(\tau ^{-1}(t'))$.

In the decomposition $L_\mathrm {FW}= B[{\mathcal {R}}_\mathrm {TW}(t)\varvec{v}_t] R_\mathrm {TW}(t)$, the rotation is applied before the Lorentz boost, hence it represents the rotation of the axes of a frame whose origin is at rest in the lab frame, and whose axes are rotating relative to the lab frame. The rotation rate, as measured in the lab frame, is the skew-symmetric matrix $\Omega$ defined by $\frac{d}{dt}{\mathcal {R}}_\mathrm {TW}(t)= \Omega (t){\mathcal {R}}_\mathrm {TW}(t)$. The two rotation rates satisfy $\Omega (t)=\Omega '(\tau (t))/\gamma (t)$.

The relation between the proper time $t'$ of the accelerated particle $O'$ and the time *t* of the lab is given, up to arbitrary choices of initial time, by the well known time dilation formula $d t'/d t = \gamma ^{-1}_t$, where $\gamma _t=[1-v^2_t]^{-1/2}$ is the Lorentz contraction factor. For a given motion of $O'$, one finds, after integrating, $t'=\tau (t)$, and upon inversion $t=\tau ^{-1}(t')$. Relativistic invariant formulas are independent of the reference frame, hence they are expressed in terms of proper time. We will use the time dilation formula and pass to the inertial time *t* when representing the formulas in the lab frame, as we did in the preceding paragraph. Sometimes, we will write equations depending on $t'$ on one side and on *t* on the other side. In these cases, for brevity, we will sometimes omit to write $t=\tau ^{-1}(t')$ or $t'=\tau (t)$.

The 3-vector corresponding to the skew-symmetric matrix $\Omega '_{t'}=\boldsymbol{\omega }'^*_{t'}{\boldsymbol{\cdot }}\varvec{{\mathcal {J}}}$, with $\varvec{{\mathcal {J}}}$ the three $(3\times 3)$ generators of rotations in 3D, is

31 $$\begin{aligned} \boldsymbol{\omega }'^*_{t'}= \frac{\gamma _{t'}}{\gamma _{t'}+1} \varvec{v}'^*_{t'}\boldsymbol{\times }\varvec{a}'^*_{t'}=-\frac{\gamma ^3_{t}}{\gamma _{t}+1} \varvec{v}_{t}\boldsymbol{\times }\varvec{a}_{t}, \end{aligned}$$

where $\varvec{v}'^*_{t'}$ is the velocity of the lab frame relative to the boosted frame, $\gamma _{t'}$ is the corresponding Lorentz factor, and $\varvec{a}'^*_{t'}$ is the proper acceleration of the boosted frame. The second equality specifies the rotation in terms of quantities measured in the lab frame.

The vector associated to the skew-symmetric matrix $\Omega _t$ is

32 $$\begin{aligned} \boldsymbol{\omega }_{t}= -\frac{\gamma ^2_{t}}{\gamma _{t}+1} \varvec{v}_{t}\boldsymbol{\times }\varvec{a}_{t}. \end{aligned}$$

We recognize in Eq. (32) the accepted value for the Thomas precession: it is not, therefore, an additional rotation of the spin in the lab frame, but rather the rotation of an auxiliary purely rotating reference frame.

### Equation of motion for the spin

#### Invariant equation

The fundamental invariant equation for $\underline{S}$ must be determined. In the following, a double underline indicates a rank-2 tensor, e.g. $\underline{\underline{F}}$. A rank-2 tensor accepts two vectorial arguments, one to the left and one to the right. By juxtaposing two vectors $\underline{A}\underline{B}$ we mean a rank-2 tensor which can be multiplied to the left or to the right, e.g. if $\underline{\underline{C}}=\underline{A}\underline{B}$, $\underline{V}{\boldsymbol{\cdot }}\underline{\underline{C}}=\underline{V} {\boldsymbol{\cdot }}\underline{A} \underline{B}$, $\underline{\underline{C}}{\boldsymbol{\cdot }}\underline{V}=\underline{A} \underline{B}{\boldsymbol{\cdot }}\underline{V}$.

Assuming a linear first order equation for a 4-vector $\underline{S}$, its motion is necessarily of the form ($\partial _{t'} = d/d{t'}$)

33 $$\begin{aligned} \partial _{t'}\underline{S}_{t'} = \lambda \underline{\underline{\Phi }}_{t'}{\boldsymbol{\cdot }}\underline{S}_{t'}+\underline{\underline{{\mathfrak {B}}}}_{t'}{\boldsymbol{\cdot }}\underline{S}_{t'} , \end{aligned}$$

with $\lambda$ a coupling constant, $\underline{\underline{\Phi }}_{t'}$ an applied tensor field and $\underline{\underline{{\mathfrak {B}}}}_{t'}:= \underline{U}_{t'} \underline{A}_{t'}-\underline{A}_{t'} \underline{U}_{t'}$ the generator of FW transport, $\underline{U}_{t'}$ being the 4-velocity of $O'$ and $\underline{A}_{t'}:=\partial _{t'} \underline{U}_{t'}$ being its 4-acceleration.

The symbol $\underline{\underline{{\mathfrak {B}}}}$ for $\underline{U}\wedge \underline{A}$ was chosen to stress that the FW generator is the Minkowski space analog of the unnormalized binormal vector of the Frenet–Serret apparatus in 3-dimensional space, $\kappa {\hat{\varvec{b}}} = {\hat{\varvec{v}}}\boldsymbol{\times }{\hat{\varvec{n}}}$, $\kappa$ being the curvature and ${\hat{\varvec{n}}}=\frac{d{\hat{\varvec{v}}}}{dl}$ the first normal. In Minkowski space, the unit tangent vector ${\hat{\varvec{v}}}$ corresponds to the 4-velocity $\underline{U}$, the curvature to the norm of the 4-acceleration $\underline{A}$, the first normal ${\hat{\varvec{n}}}$ to the direction of the 4-acceleration $\underline{A}$, and the curve length parameter *l* to the proper time.

For a spin 1/2 interacting with the electromagnetic field, $\lambda$ is the gyromagnetic factor, i.e. the constant of proportionality between the spin and the intrinsic magnetic moment, which is the Bohr magneton times the Landé $g_\mathrm {L}$ factor divided by the total angular momentum, $\lambda = g_\mathrm {L} q/(2m)$. The skew-symmetry of $\underline{\underline{{\mathfrak {B}}}}$ ensures the conservation of the norm of $\underline{S}$ in absence of external fields. The first term on the rhs of Eq. (33) would be zero in the absence of fields acting directly on $\underline{S}$. Hence, we shall refer to the terms arising from it as the direct part.

If, furthermore, the field is $\underline{\underline{\Phi }}_{t'}= \underline{\underline{\Pi }}_{t'}{\boldsymbol{\cdot }}\underline{\underline{F}}_{t'}{\boldsymbol{\cdot }}\underline{\underline{\Pi }}_{t'}$, with $\underline{\underline{F}}_{t'}$ skew-symmetric and $\underline{\underline{\Pi }}_{t'}=1+\underline{U}_{t'}\underline{U}_{t'}$, the projection operator on the three-dimensional space normal to the 4-velocity $\underline{U}_{t'}$, then Eq. (33) preserves both the norm of $\underline{S}$ and the value of its projection along the worldline $O'$, $\underline{S}_{t'}{\boldsymbol{\cdot }}\underline{S}_{t'}=const$ and $\underline{U}_{t'}{\boldsymbol{\cdot }}\underline{S}_{t'}=const$. In particular, when the constant projection is zero, the field $\underline{\underline{F}}$ is the electromagnetic field, and the acceleration is due solely to $\underline{\underline{F}}$ (neglecting the Stern–Gerlach contribution to the acceleration^24^) $m\underline{A}_{t'}=q\underline{\underline{F}}_{t'}{\boldsymbol{\cdot }}\underline{U}_{t'}$, then Eq. (33) reduces to the Bargmann–Michel–Telegdi equation^9^.

#### Equation for components in an arbitrary frame

A reference frame is characterized by an origin *O* following a given worldline in Minkowski space, and by a family of three vectors $\underline{e}_{(j)}(t)$ normal to the worldline, the spatial axes. (To avoid confusion, we are writing the time dependence in conventional form, $\underline{e}_{(j)}(t), S^j(t)$ when indexes appear. In the following, however, we will avoid as far as possible the use of component indexes. All in all, special relativity is a geometric theory, and thus it is possible to formulate its equations in an invariant form.)

The spatial axes are transported along the worldline of the origin of the frame in such a way that the angles among them are preserved and that they continue normal to the time axis, the 4-velocity, which implies that $\partial _{t}\underline{e}_{(j)}(t)=\underline{\underline{{\mathfrak {B}}}}^{(Frame)}_{t}{\boldsymbol{\cdot }}\underline{e}_{(j)}(t) +\Omega ^k_{\ j}(t)\underline{e}_{(k)}(t)$ with $\Omega ^{j}_{\ k}= \Omega ^{j k'}\underline{e}_{(k')}{\boldsymbol{\cdot }}\underline{e}_{(k)}$, $\Omega ^{j k}$ a skew-symmetric $3\times 3$ matrix and *t* the proper time of the frame. The tensor $\underline{\underline{{\mathfrak {B}}}}^{(Frame)}=\underline{U}^{(Frame)}\wedge \frac{d}{dt}\underline{U}^{(Frame)}$ is the FW transport generator associated to the frame, while the tensor $\underline{\underline{{\mathfrak {B}}}}$ that appears in Eq. (33) is the one associated to the particle.

The spin vector is to be decomposed not in the basis $\underline{e}_{(\alpha )}(t)$ at the origin, but in another basis $\underline{e}_{(\alpha )}(\varvec{r}_t,t)$ depending on the position of the spin. The two bases differ because in an accelerated reference frame a curvature appears. Details of the calculations will be shown in a forthcoming paper^25^.

The components of the spin 4-vector are $S^\alpha (t)=\underline{S}(t'){\boldsymbol{\cdot }}\underline{e}^{(\alpha )}(\varvec{r}_t,t)$, where $\underline{e}^{(\alpha )}(\varvec{r}_t,t)$ are the reciprocal spatial axes of the frame, $\underline{e}^{(\alpha )}(\varvec{r}_t,t){\boldsymbol{\cdot }}\underline{e}_{(\beta )}(\varvec{r}_t,t)=\delta ^\alpha _{\ \beta }$.

Thus

34 $$\begin{aligned} \partial _{t} S^\alpha (t)&= \frac{1}{{\bar{\gamma }}_t} \left( \partial _{t'}\underline{S}_{t'}\right) {\boldsymbol{\cdot }}\underline{e}^{(\alpha )}(\varvec{r}_t,t) +\underline{S}_{t'}{\boldsymbol{\cdot }}\left( \partial _t\underline{e}^{(\alpha )}(\varvec{r}_t,t)\right) \end{aligned}$$

The factor ${\bar{\gamma }}_t=[\partial t'/\partial t]^{-1}$ is the time dilation, which in an accelerated frame is

35 $$\begin{aligned} {\bar{\gamma }}_t = \frac{1}{\sqrt{(1-\varvec{g}_t{\boldsymbol{\cdot }}\varvec{r}_t)^2-[\varvec{v}_t-\boldsymbol{\Omega }_t\times \varvec{r}_t]^2}}, \end{aligned}$$

$\varvec{g}$ being the gravitational field at the origin of the frame due to the equivalence principle, i.e. minus the proper acceleration, $\boldsymbol{\Omega }$ the Coriolis gravi-magnetic field, i.e. the angular velocity of the axes of a FW frame as measured by the accelerated frame, and $\varvec{r}_t$ the position of the particle. We shall define the scalar potential $\theta _t=1-\varvec{g}_t{\boldsymbol{\cdot }}\varvec{r}_t$ and the vector potential $\varvec{w}_t=\boldsymbol{\Omega }_t\times \varvec{r}_t$.

The additional terms due to the second addend on the rhs of Eq. (34) lead to a redefinition of the time derivative as a tensor operator,

36 $$\begin{aligned} D^\alpha _{\ \beta }=\delta ^\alpha _{\ \beta }\partial _t - \left( \frac{d}{dt}\underline{e}^{(\alpha )}(\varvec{r}_t,t)\right) {\boldsymbol{\cdot }}\underline{e}_{(\beta )}(\varvec{r}_t,t) , \end{aligned}$$

so that the laws of physics are formally invariant even when written in terms of components. By contrast, the formulation of the preceding subsection in terms of abstract vectors and tensors does not require any redefinition of derivatives.

After some calculations, we find that the spatial components of the spin 4-vector obey

37 $$\begin{aligned} \partial _t \varvec{S}_t&= \left[ \frac{\lambda }{{\bar{\gamma }}_t}{\mathcal {E}}_t+{\mathcal {K}}_t+{\mathcal {N}}_t\right]\varvec{S}_t\ , \end{aligned}$$

where the matrices ${\mathcal {E}}$, ${\mathcal {K}}$, and ${\mathcal {N}}$ represent, respectively, the electromagnetic, the kinematic, and the non inertial contribution,

38a $$\begin{aligned} {\mathcal {E}}_t&= -\left[ \mathbbm {1}_3+{\bar{\gamma }}^2_t\varvec{v}_t\varvec{v}^\mathrm {tr}_t-\frac{1}{\theta _t^2}\varvec{w}_t\varvec{w}^\mathrm {tr}_t\right] \left( \varvec{B}_t-\varvec{v}^\mathrm {c}_t{\boldsymbol{\times }}\varvec{E}_t\right) {\boldsymbol{\cdot }}\varvec{{\mathcal {J}}} , \end{aligned}$$

38b $$\begin{aligned} {\mathcal {K}}_t&=\varvec{v}_t \varvec{A}^{\mathrm {sim},\mathrm {tr}}_t{\boldsymbol{\cdot }}\left[ \mathbbm {1}_3-\varvec{w}_t\varvec{v}^{\mathrm {c},\mathrm {tr}}_t\right] , \end{aligned}$$

38c $$\begin{aligned} {\mathcal {N}}_t&= \left[ \theta _t\varvec{g}_t+\partial _t\varvec{w}_t -\frac{1}{\theta _t} (\partial _t\theta _t+\varvec{w}_t{\boldsymbol{\cdot }}\varvec{g}_t)\varvec{w}_t -\boldsymbol{\Omega }_t\times \varvec{w}_t\right] \varvec{v}_t^{\mathrm {c},\mathrm {tr}} +\boldsymbol{\Omega }_t{\boldsymbol{\cdot }}\varvec{{\mathcal {J}}} +\frac{1}{\theta _t} \varvec{w}_t\varvec{g}_t^\mathrm {tr}, \end{aligned}$$

$\mathbbm {1}_3$ being the $3\times 3$ identity and $\varvec{a}\varvec{b}^\mathrm {tr}$ being a matrix written as a dyadic. The symbol $\varvec{{\mathcal {J}}}$ denotes the 3-vector having as components the $3\times 3$ generators of spatial rotations, such that for any pair $\varvec{a}, \varvec{b}$ of 3-vectors $\varvec{a}{\boldsymbol{\cdot }}\varvec{{\mathcal {J}}}\, \varvec{b}=\varvec{a}\boldsymbol{\times }\varvec{b}$ and $\varvec{b}^\mathrm {tr}\varvec{a}{\boldsymbol{\cdot }}\varvec{{\mathcal {J}}} =(\varvec{b}\boldsymbol{\times }\varvec{a})^\mathrm {tr}$. We introduced the simultaneous 3-acceleration $\varvec{A}^\mathrm {sim}=\varvec{A}-A^0\varvec{v}$, $A^0=\varvec{v}^\mathrm {c}{\boldsymbol{\cdot }}\varvec{A}$, and the conjugate velocity

39 $$\begin{aligned} \varvec{v}^\mathrm {c}_t=\frac{\varvec{v}_t-\varvec{w}_t}{\theta _t^2-\varvec{w}_t^2+\varvec{w}_t{\boldsymbol{\cdot }}\varvec{v}_t} . \end{aligned}$$

The time component satisfies identically $S^0(t) = \varvec{v}^\mathrm {c}_t{\boldsymbol{\cdot }}\varvec{S}_t$.

In order to identify correctly the electric and magnetic field in an accelerated frame, some care must be taken because the metric is not flat. One should use the fully covariant representation

40 $$\begin{aligned} \underline{e}_{(\alpha )}(\varvec{r}_t,t){\boldsymbol{\cdot }}\underline{\underline{F}}{\boldsymbol{\cdot }}\underline{e}_{(\beta )}(\varvec{r}_t,t) = \begin{pmatrix}0&{}-\varvec{E}^\mathrm {tr}\\ \varvec{E}&{}-\varvec{B}{\boldsymbol{\cdot }}\varvec{{\mathcal {J}}}\end{pmatrix} \ . \end{aligned}$$

In a nonrotating frame, i.e. a FW frame, $\boldsymbol{\Omega }=0$, and the equivalent gravitational field is Newtonian, as it derives from a scalar potential, hence

41 $$\begin{aligned} \partial _t \varvec{S}_t&= -\frac{\lambda }{{\bar{\gamma }}_t}\left[ \mathbbm {1}_3+{\bar{\gamma }}^2_t\varvec{v}_t\varvec{v}^\mathrm {tr}_t\right] \left[ \left( \varvec{B}_t-\frac{\varvec{v}_t}{\theta _t^2}{\boldsymbol{\times }}\varvec{E}_t\right) \boldsymbol{\times }\varvec{S}_t\right] +\varvec{v}_t \varvec{A}^{\mathrm {sim}}_t{\boldsymbol{\cdot }}\varvec{S}_t+\frac{\varvec{g}_t}{\theta _t}\varvec{v}_t{\boldsymbol{\cdot }}\varvec{S}_t . \end{aligned}$$

#### Equation in a comoving frame

In the following, a prime with an asterisk denotes the representation in a comoving frame, while a prime denotes the representation in the FW comoving frame.

In a comoving frame $\varvec{v}=\varvec{0}$ and $\varvec{r}=\varvec{0}$, thus $\theta =1$, $\varvec{w}=\varvec{0}$, and Eq. (37) simplifies to

42 $$\begin{aligned} \partial _{t'}{\varvec{S}}'^*_{t'} = {\varvec{S}}'^*_{t'}\boldsymbol{\times }[\lambda {\varvec{B}}'^*_{t'} -\boldsymbol{\Omega }'^*_{t'}] , \end{aligned}$$

where ${\varvec{B}}'^*_{t'}$ is the magnetic field in the comoving frame, which is the axial 3D-vector representation of the spatial block of the skew-symmetric tensor $\underline{\underline{\Pi }}_{t'}{\boldsymbol{\cdot }}\underline{\underline{F}}_{t'}{\boldsymbol{\cdot }}\underline{\underline{\Pi }}_{t'}$.

For FW frames $\boldsymbol{\Omega }'=\varvec{0}$. Thus, Eq. (33) becomes particularly simple in the comoving FW frame:

43 $$\begin{aligned} \partial _{t'}{\varvec{S}}'_{t'} = \lambda {\varvec{S}}'_{t'}\boldsymbol{\times }{\varvec{B}}'_{t'} . \end{aligned}$$

The electromagnetic field felt by the particle $\varvec{E}', \varvec{B}'$ is not given by the standard textbook formulas $\varvec{E}_\parallel +\gamma (\varvec{E}_\perp +\varvec{v}\boldsymbol{\times }\varvec{B})$, $\varvec{B}_\parallel +\gamma (\varvec{B}_\perp -\varvec{v}\boldsymbol{\times }\varvec{E})$, because the Lorentz transformation $L_t$ from the lab frame to the comoving frame is not a simple boost, but includes the Thomas–Wigner rotation. See Eq. (15) for an example.

#### Equation in an inertial frame

In an inertial frame, the axes do not vary. In particular, $\varvec{g}=\varvec{0}$ and $\boldsymbol{\Omega }=\varvec{0}$. Hence, Eq. (37) simplifies to

44a $$\begin{aligned} \partial _t \varvec{S}_t&= {\mathcal {M}}(t)\varvec{S}_t\ , \end{aligned}$$

44b $$\begin{aligned} \partial _t \varvec{T}_t&= -{\mathcal {M}}^\mathrm {tr}(t)\varvec{T}_t \ . \end{aligned}$$

where the matrix ${\mathcal {M}}$ is

45 $$\begin{aligned} {\mathcal {M}}(t)&:=-\left[ \mathbbm {1}_3+\gamma ^2_t\varvec{v}_t\varvec{v}^\mathrm {tr}_t\right] \left[ \lambda \gamma ^{-1}_t \left( \varvec{B}_t-\varvec{v}_t{\boldsymbol{\times }}\varvec{E}_t\right) {\boldsymbol{\cdot }}\varvec{{\mathcal {J}}} -\varvec{v}_t\varvec{a}^\mathrm {tr}_t\right] , \end{aligned}$$

The time component satisfies identically $S^0(t) = \varvec{v}_t{\boldsymbol{\cdot }}\varvec{S}_t$. The terms on the rhs multiplied by $\lambda$ are the direct terms, the remaining term $\left[ \mathbbm {1}_3+\gamma ^2_t\varvec{v}_t\varvec{v}^\mathrm {tr}_t\right] \varvec{v}_t\varvec{a}^\mathrm {tr}_t= \gamma ^2_t \varvec{v}_t\varvec{a}^\mathrm {tr}_t$ for $\varvec{S}$, or $-\gamma ^2_t \varvec{a}_t\varvec{v}^\mathrm {tr}_t$ for $\varvec{T}$, is the kinematic term.

### Separation of norm and direction in the motion of a vector

Here we discuss the linear homogeneous equation for an *N*-dimensional vector

46 $$\begin{aligned} {\partial _t \varvec{P}(t)}= M(t)\varvec{P}(t)\ , \end{aligned}$$

with *M*(*t*) a given one-parameter family of matrices.

We are indicating with a bold symbol a column vector. A row vector will be indicated as a transpose, e.g. $\varvec{P}^\mathrm {tr}$. The row-column product is implied by juxtaposition of the symbols. Thus, $\varvec{a}^\mathrm {tr}\varvec{b}$ is the scalar $\varvec{a}{\boldsymbol{\cdot }}\varvec{b}$, while $\varvec{a}\varvec{b}^\mathrm {tr}$ is a matrix; $M\varvec{P}$ is a column vector, $\varvec{P}^\mathrm {tr}M$ a row vector, and $\varvec{P}^\mathrm {tr}M\varvec{P}$ is a scalar.

If *M*(*t*) is skew-symmetric, the norm of $\varvec{P}(t)$ is constant, thus the equation describes a rotation. However, how to proceed if *M*(*t*) is not skew-symmetric? Can we still individuate a rotation term?

First, we shall drop from the problem any component which is possibly conserved and does not enter the equations for the other components. This happens if *M*(*t*) has some rows and the corresponding columns identically equal to zero. Thus, if *M* can be written in the block form $$({\begin{matrix}M_0(t)&{}0\\ 0&{}0\end{matrix}})$$, we shall consider only the block $M_0$ and the corresponding components of $\varvec{P}$, since the remaining components are constant.

More generally, if *M*(*t*) has a set of $\mu$ common, constant in *t*, left and right eigenvectors $\varvec{e}_m$ with 0 eigenvalue, we change coordinates so that the last $\mu$ axes coincide with $\varvec{e}_m$. Then, since $\varvec{P}(t)\cdot \varvec{e}_m=const$, we consider only the remaining components of $\varvec{P}$, and the corresponding non-zero block of *M*.

Next, we separate *M*(*t*), or its relevant part $M_0(t)$, in a symmetric and a skew-symmetric part, $M(t)={\overline{M}}(t)+M_\mathrm {a}(t)$. The skew-symmetric part gives automatically a contribution perpendicular to $\varvec{P}$, hence it provides a rotation. The symmetric part, however, also provides a rotation, albeit a nonlinear one, as we shall see in the following.

We write the vector as $\varvec{P}(t)=P(t){\hat{\varvec{P}}}(t)$, and we separate the equations for the norm *P*(*t*) and for the direction ${\hat{\varvec{P}}}(t)$. By construction, the equation for the direction can be only a rotation. By multiplying Eq. (46) with $\varvec{P}(t)$, we get the equation for the norm, which involves only the symmetric part of *M*,

47 $$\begin{aligned} P(t)\frac{\partial {P}(t)}{\partial t} = P^2(t)\, {\hat{\varvec{P}}}^\mathrm {tr}(t) {\overline{M}}(t){\hat{\varvec{P}}}(t)\ . \end{aligned}$$

The direction instead satisfies

48 $$\begin{aligned} \frac{\partial {\hat{\varvec{P}}}(t)}{\partial t} = [{\overline{M}}(t)-{\hat{\varvec{P}}}^\mathrm {tr}(t) {\overline{M}}(t) {\hat{\varvec{P}}}(t)]{\hat{\varvec{P}}}(t)+M_\mathrm {a}(t){\hat{\varvec{P}}}(t)\ . \end{aligned}$$

The first term in the rhs can be written

49 $$\begin{aligned}{}[{\overline{M}}(t)-{\hat{\varvec{P}}}^\mathrm {tr}(t) {\overline{M}}(t) {\hat{\varvec{P}}}(t)]{\hat{\varvec{P}}}(t)= A_\mathrm {nl}(t,\hat{\varvec{P}}(t)){\hat{\varvec{P}}}(t) \ , \end{aligned}$$

where we defined the skew-symmetric matrix

50 $$\begin{aligned} A_\mathrm {nl}(t,\hat{\varvec{P}}):=[{\overline{M}}(t),{\hat{\varvec{P}}}{\hat{\varvec{P}}}^\mathrm {tr}] \ , \end{aligned}$$

with $[\ ,\ ]$ the commutator. The skew-symmetric matrix $A_\mathrm {nl}(t,\hat{\varvec{P}})$ hence corresponds to another rotation, which is nonlinear, as it depends on the direction ${\hat{\varvec{P}}}$ itself.

From Eq. (47), the square norm can be written after possibly solving for the direction,

51 $$\begin{aligned} P^2(t)=P^2(0)\,\exp {[\,2\!\int _0^t dt\, {\hat{\varvec{P}}}^\mathrm {tr}(t) {\overline{M}}(t){\hat{\varvec{P}}}(t)]}. \end{aligned}$$

#### Three dimensional case

In particular, in three dimensions, to any skew-symmetric matrix it is associated, through the Hodge dual operation, an axial vector which can be identified as an angular velocity. The linear angular velocity is

52 $$\begin{aligned} \boldsymbol{\omega }^\mathrm {l}(t){\boldsymbol{\cdot }}\varvec{{\mathcal {J}}} = M_\mathrm {a}(t) \ , \end{aligned}$$

with $\varvec{{\mathcal {J}}}$ the $3\times 3$ angular momentum generators, which form a basis for skew-symmetric matrices. The nonlinear angular velocity satisfies

53 $$\begin{aligned} \boldsymbol{\omega }^\mathrm {nl}(t,{\hat{\varvec{P}}}){\boldsymbol{\cdot }}\varvec{{\mathcal {J}}} =A_\mathrm {nl}(t,\hat{\varvec{P}}) . \end{aligned}$$

Explicitly,

54 $$\begin{aligned} \boldsymbol{\omega }^\mathrm {nl}(t,{\hat{\varvec{P}}})={\hat{\varvec{P}}}\boldsymbol{\times }\left( {\overline{M}}(t){\hat{\varvec{P}}}\right) \ . \end{aligned}$$

Finally, in three dimensions, the equations for the direction can be separated as

55 $$\begin{aligned} \frac{\partial {\hat{\varvec{P}}}(t)}{\partial t} =[\boldsymbol{\omega }^\mathrm {l}(t)+ \boldsymbol{\omega }^\mathrm {nl}(t,{\hat{\varvec{P}}}(t)) ]\boldsymbol{\times }{\hat{\varvec{P}}}(t). \end{aligned}$$

#### Two-dimensional case

Suppose that the *Z* component of the vector is conserved, and that it does not enter in the equation for the other two components. This case is relevant because often times the trajectory is planar, and the off-plane component of the spin is always conserved. Let $M_{\mathrm{II}}$ the $2\times 2$ nonzero block of *M*. Let $\varvec{P}_\parallel =\{\varvec{P}_{\mathrm{II}},0\}$ the component of $\varvec{P}$ on the *XY* plane with $\varvec{P}_{\mathrm{II}}$ the 2-component vector consisting in the first two components of $\varvec{P}$. We have an effective two-dimensional equation

56 $$\begin{aligned} \partial _t \varvec{P}_{\mathrm{II}}(t)=M_{\mathrm{II}}(t) \varvec{P}_{\mathrm{II}}(t) . \end{aligned}$$

We may proceed as in the general case, and apply Eq. (50) to $\varvec{P}_{\mathrm{II}}(t)$. Since there is only one skew-symmetric matrix in 2D, up to a constant, $$A_Z=\left({\begin{matrix}0&{}-1\\ 1&{}0\end{matrix}}\right)$$, the commutator yields

57 $$\begin{aligned} A_\mathrm {nl}(t,\hat{\varvec{P}}_\parallel ):=[{\overline{M}}_{\mathrm{II}}(t),{\hat{\varvec{P}}}_{\mathrm{II}}{\hat{\varvec{P}}}_{\mathrm{II}}^\mathrm {tr}]=\omega ^\mathrm {nl}(t,{\hat{\varvec{P}}}_\parallel (t)) A_Z, \end{aligned}$$

where $\omega ^\mathrm {nl}(t,{\hat{\varvec{P}}}_\parallel )={\hat{\varvec{k}}}{\boldsymbol{\cdot }}[{\hat{\varvec{P}}}_\parallel (t)\boldsymbol{\times }({\overline{M}}(t){\hat{\varvec{P}}}_\parallel (t))]$, while ${\hat{\varvec{P}}}_\parallel (t)$ is the unit vector of the component of $\varvec{P}$ parallel to the *XY* plane, i.e. if $\varvec{P}=\{X,Y,Z\}$, ${\hat{\varvec{P}}}_\parallel =\{X,Y,0\}/\sqrt{X^2+Y^2}$. Better, in invariant form $|\varvec{P}_\parallel |=|\varvec{P}\boldsymbol{\times }{\hat{\varvec{k}}}|$, ${\hat{\varvec{P}}}_\parallel =(\varvec{P}-{\hat{\varvec{k}}}{\boldsymbol{\cdot }}\varvec{P}\, {\hat{\varvec{k}}})/|\varvec{P}_\parallel |$.

The nonlinear rotation rate, as a vector in 3D, is thus

58 $$\begin{aligned} \boldsymbol{\omega }^\mathrm {nl}(t,{\hat{\varvec{P}}}) = \frac{\{{\hat{\varvec{P}}}\boldsymbol{\times }[{\overline{M}}(t){\hat{\varvec{P}}}]\}{\boldsymbol{\cdot }}{\hat{\varvec{k}}}\, {\hat{\varvec{k}}}}{|{\hat{\varvec{P}}}\times {\hat{\varvec{k}}}|^2} \end{aligned}$$

Had we not eliminated from the equations the constant component *Z*, and had we instead applied the result for the 3D case, we would get in-plane terms in $\boldsymbol{\omega }^\mathrm {nl}$, which would change the *Z* component of the unit vector ${\hat{\varvec{P}}}$ in such a way as to compensate the change in the norm *P*.

#### Application to the spin precession

In our case, in absence of external fields, the equation for the simultaneity-corrected spin in an inertial frame is

59 $$\begin{aligned} \frac{\partial }{\partial t} \varvec{T}(t) = -\gamma ^2(t)\varvec{a}(t) \varvec{v}(t){\boldsymbol{\cdot }}\varvec{T}(t), \end{aligned}$$

hence the matrix is

60 $$\begin{aligned} M(t) = -\gamma ^2(t)\varvec{a}(t) \varvec{v}^\mathrm {tr}(t) . \end{aligned}$$

The symmetric part is

61 $$\begin{aligned} {\overline{M}}(t)=-\frac{1}{2}\gamma ^2(t)\frac{d}{dt}\varvec{v}(t)\varvec{v}^\mathrm {tr}(t)\ . \end{aligned}$$

Therefore, the nonlinear part of the precession (since a spin represents a rotation, the rotation of a spin is called a precession),

62 $$\begin{aligned} \boldsymbol{\omega }^\mathrm {nl}(t,{\hat{\varvec{T}}})&= -\frac{\gamma ^2(t)}{2}{\hat{\varvec{T}}}\boldsymbol{\times }\left\{ \left[ \frac{d}{dt}\varvec{v}(t) \varvec{v}^\mathrm {tr}(t)\right] {\hat{\varvec{T}}}\right\} \ . \end{aligned}$$

However, if the motion is planar, the component of the spin normal to the plane of motion in the lab frame is conserved. Thus, we should use the 2D result, which yields a nonlinear rotation rate

63 $$\begin{aligned} \boldsymbol{\omega }^\mathrm {nl}(t,{\hat{\varvec{T}}})&= -\frac{\gamma ^2(t)}{2}\left( {\hat{\varvec{T}}}_\parallel \boldsymbol{\times }\left\{ \left[ \frac{d}{dt}\varvec{v}(t) \varvec{v}^\mathrm {tr}(t)\right] {\hat{\varvec{T}}}_\parallel \right\} \right) {\boldsymbol{\cdot }}{\hat{\varvec{k}}}\, {\hat{\varvec{k}}}\ . \end{aligned}$$

The skew-symmetric term is

64 $$\begin{aligned} M_\mathrm {a}=-\frac{1}{2}\gamma ^2(t)[\varvec{a}(t)\varvec{v}^\mathrm {tr}(t)-\varvec{v}(t)\varvec{a}^\mathrm {tr}(t)]\ , \end{aligned}$$

corresponding to the Thomas precession

65 $$\begin{aligned} \boldsymbol{\omega }^\mathrm {Th}(t)= -\frac{\gamma ^2(t)}{2}\varvec{v}(t)\boldsymbol{\times }\varvec{a}(t) \ . \end{aligned}$$

For the conventional spin $\varvec{S}$ the same linear term holds, while the non-linear term has the opposite sign.

## Supplementary Information

Supplementary Information 1.

Supplementary Information 2.

Supplementary Information 3.

Supplementary Information 4.

Supplementary Information 5.

## Data Availability

All data generated or analysed during this study are included in this published article (and its Supplementary Information files).
