# Supplementary material for: Nonlinear effects in Thomas precession due to the interplay of Lorentz contraction and Thomas–Wigner rotation
Source: Sci Rep. 2022 Oct 6;12:16757. doi: 10.1038/s41598-022-20942-w (PMC9537424; doi:10.1038/s41598-022-20942-w)
Supplement: Supplementary file 1 — Supplementary Information 1. [file 41598_2022_20942_MOESM1_ESM.pdf]

Supplemental materials to Nonlinear effects in Thomas precession due to the interplay of Lorentz contraction and Thomas–Wigner rotation

Antonio Di Lorenzo

Instituto de Física, Universidade Federal de Uberlândia, Av. João Naves de Ávila 2121, Uberlândia, Minas Gerais, 38400-902, Brazil

A An overview of the notation and known results

A.1 Notation

We will use an underscore for abstract vectors in Minkowski space, while an arrow will indicate a contravariant representation of the vector in some reference frame, which we define below. Greek labels range in $\{0,1,2,3\}$, while Latin labels range in $\{1,2,3\}$. The convention of summing over repeated indexes is used, so that $u^j v_j = \sum_{j=1}^{3} u^j v_j$, $u^\alpha v_\alpha = \sum_{\alpha=0}^{3} u^\alpha v_\alpha$. However, as soon as possible, we shall replace sums over components with dot products, for ease of readability. The signature of the metric is chosen as $-{+}{+}{+}$.

Observers are represented by worldlines in Minkowski spacetime, usually indicated by a letter O, primed or not. The worldline can be parametrized with its natural parameter, the proper time τ_O or t for short. A reference frame $\mathfrak{S}$ consists in a worldline O, along with a set of 4 vectors, $\underline{e}_{(\alpha)}(t)$, such that the first one $\underline{e}_{(0)}(t)$ is the unit tangent vector to the worldline ($\underline{e}_{(0)}(t){\cdot}\underline{e}_{(0)}(t) = -1$), while the other three $\underline{e}_{(j)}(t)$ are orthogonal to $\underline{e}_{(0)}(t)$. The latter three are the spatial axes of the reference frame. While they need not be mutually orthogonal, their relative angles are required to be fixed, so that the metric, defined by $g_{\alpha\beta} := \underline{e}_{(\alpha)}(t){\cdot}\underline{e}_{(\beta)}(t)$, is constant along the worldline, and furthermore has the block representation $g = \left(\begin{smallmatrix} -1 & \mathbf{0} \\ \mathbf{0} & \mathcal{G}\end{smallmatrix}\right)$, with $\mathcal{G}$ a constant 3×3 matrix.

The metric allows to define the reciprocal vectors $\underline{e}^{(\alpha)}(t) = (g^{-1})^{\alpha\beta}\underline{e}_{(\beta)}(t)$, with g^{-1} the inverse of g. Thus $\underline{e}^{(0)} = -\underline{e}_{(0)}$. One may want to choose $\mathcal{G}$ to be the 3×3 identity, by picking an orthonormal basis, in which case $\underline{e}^{(j)} = \underline{e}_{(j)}$. The corresponding matrix is then the Minkowski metric $g = g^{-1} = \eta$. This choice, however is not cogent: the laws of Nature are indifferent to the fact that we find easier expressing them in an orthonormal basis. The orthogonality between the

time axis $\underline{e}_{(0)}$ and the spatial axes $\underline{e}_{(j)}$, on the other hand, is fundamental, not an arbitrary choice.

A.2 Contravariant and covariant vector representations

In a given basis, the components of a vector transported along the same worldline, $\underline{V}(t) = V^\alpha(t)\underline{e}_{(\alpha)}(t)$ are obtained by multiplying times the reciprocal basis $V^\alpha(t) = \underline{V}(t)\cdot\underline{e}^{(\alpha)}(t)$. These components, collected in a column vector, will be denoted by the symbol $\vec{V}$, which is the representation of $\underline{V}$ in the reference frame defined by the basis $\underline{e}_{(\alpha)}$. For increased readability, a column vector will be written as a horizontal list in braces $\{V^0, V^1, V^2, V^3\}$. One may want to write the components in the reciprocal basis, which will be denoted by a lower index, $V_\alpha := \underline{V}\cdot\underline{e}_{(\alpha)}$. In this case we shall enclose the list in round parentheses, (V_0, V_1, V_2, V_3), consider the vector as a row vector, and indicate it as $\vec{V}^{\mathrm{c}}$. Given the metric representation g, $\vec{V}^{\mathrm{c}} = [g\cdot\vec{V}]^{\mathrm{tr}}$. Furthermore $V_0 = -V^0$, while the spatial components are $(V_1, V_2, V_3) = [\mathcal{G}\cdot\boldsymbol{V}]^{\mathrm{tr}}$. In case one is using an orthonormal spatial basis, $V_j = V^j$.

In a given representation, we shall indicate column 3–vectors by boldface letters, $\boldsymbol{v}, \boldsymbol{a}$, while the row vectors will be denoted by a boldface symbol with a conjugation, $\boldsymbol{v}^{\mathrm{c}}, \boldsymbol{a}^{\mathrm{c}}$. In particular, for the Minkowski metric, since $\mathcal{G} = \mathbb{1}_3$, instead of the conjugation symbol we shall use the conventional transpose, $\boldsymbol{v}^{\mathrm{tr}}$, etc. We will also use the notation $\boldsymbol{v}\boldsymbol{a}^{\mathrm{c}}$ to indicate the direct product, a 3×3 matrix having in the j–th row and k–th column the value $v^j a_k$.

Vectors per se are invariant objects. Their components however, transform with the basis. The vector representations $\vec{U}$ and $\vec{U}^{\mathrm{c}}$ are called contravariant and covariant vectors, resp. A stricter term would be *contravariant and covariant vector representations.*

The basis in its own representation is simply the canonical basis: $\vec{e}_{(0)} = \{1, 0, 0, 0\}$, $\vec{e}_{(1)} = \{0, 1, 0, 0\}$, etc. If one considers another basis $\underline{f}_{(\alpha)}$, the former basis can be represented in it as, e.g., $\vec{e}^{\,[f]}_{(0)} = \{e^0_{(0)}, e^1_{(0)}, e^2_{(0)}, e^3_{(0)}\}$. Here, a label distinguishing a vector is indicated in parentheses, while an index with no parentheses around it denotes a component in a given basis.

A.3 Classification of reference frames

Inertial reference frames have a straight worldline O, which makes $\underline{e}_{(0)}$ a constant vector, and constant spatial axes $\underline{e}_{(j)}(t) = \underline{e}_{(j)}(0)$. A frame with constant $\underline{e}_{(0)}$ but time-varying $\underline{e}_{(j)}(t)$ is a purely rotating frame. A frame with varying time axis $\underline{e}_{(0)}(t)$ is an accelerated frame.

Among the accelerated frames sharing the same worldline O, there is a special one, the FW frame: The normality condition $\underline{e}_{(0)}(t)\cdot\underline{e}_{(j)}(t) = 0$ implies that for an accelerated frame the spatial axes must also vary in time. If we require that their time derivatives $d\underline{e}_{(j)}(t)/dt$ are parallel to $\underline{e}_{(0)}(t)$, no noninertial rotations are induced in the accelerated frame. A reference frame satisfying this condition is called a FW frame (other texts refer to it as Fermi coordinates).

Consider indeed, at a given time, a purely spatial vector representing the position of a particle E at time t relative to the observer: $E(t) - O(t)$, which means that the vector is perpendicular to the wordline O. The spatial coordinates are given by $X^j(t) = [E(t) - O(t)]\cdot\underline{e}^{(j)}(t)$. At a later time, the new coordinates of the particle are, to first order,

$$\begin{aligned} X^j(t+\delta t) &= [E(t+\delta t) - O(t+\delta t)]\cdot\underline{e}^{(j)}(t+\delta t) \\ &\simeq \left\{E(t) - O(t) + \delta t\left[\gamma_{EO}^{-1}(t)\underline{U}_E(t) - \underline{U}_O(t)\right]\right\}\cdot[\underline{e}^{(j)}(t) + \delta t\frac{d}{dt}\underline{e}^{(j)}(t)] \\ &\simeq X^j(t) + \delta t v^j_{E|O}(t) + \delta t[E(t) - O(t)]\cdot\frac{d}{dt}\underline{e}^{(j)}(t), \end{aligned} \tag{1}$$

with $\underline{U}_E$ the 4–velocity of the particle E, $\underline{U}_O(t) = \underline{e}_{(0)}(t)$ the 4–velocity of the observer O, and γ_{EO} the Lorentz dilation factor $[1-v^2_{E|O}(t)]^{-1/2}$ for the velocity of the particle E relative to O. If the derivatives $\frac{d}{dt}\underline{e}^{(j)}(t)$ contain spatial components, the last term in the rhs of the last equality will contribute a noninertial rotation to the movement of the particle as described in the accelerated reference frame. If instead they are parallel to $\underline{U}_O$, the product vanishes, because $E(t) - O(t)$ is normal to the trajectory.

This latter condition is the relativistic analog of the classical case, where, given an accelerated origin O, we may consider, among all possible comoving frames, one in which the axes are fixed relative to an inertial frame. In special relativity, however, one cannot impose that the spatial axes remain fixed $d\underline{e}^{(j)}(t)/dt = 0$, because they must be kept orthogonal to the time axis, which in an accelerated frame changes direction in Minkowski space. However, by requiring that the derivatives are parallel to the 4–velocity, one ensures that no Coriolis rotations appear in the motion of E, hence we can say that the reference frame so defined corresponds to the non–relativistic non–rotating frame.

A.4 Transformations between two frames

Upon a change of basis, the representation of a vector $\underline{V}$ transforms according to

$$V^{[f],\alpha} = \underline{V}\cdot\underline{f}^{(\alpha)} = \underline{V}\cdot\underline{e}^{(\beta)}\underline{e}_{(\beta)}\cdot\underline{f}^{(\alpha)} = T^\alpha_{\ \beta}V^{[e],\beta}, \tag{2}$$

or, more compactly,

$$\vec{V}^{[f]} = T\cdot\vec{V}^{[e]}\ . \tag{3}$$

The matrix $T^\alpha_{\ \beta} := \underline{f}^{(\alpha)}\cdot\underline{e}_{(\beta)}$ provides the change of vectors applied at the origin. The upper index indicates the rows, and the lower index the columns, so that the dot "·" symbol in Eq. (3) indicates the ordinary row-column product.

We are overloading the same symbol with different meanings, which, however, are clear from the objects to which it is applied: $\underline{A}\cdot\underline{B}$ is the product in Minkowski space, $\vec{A}^{\rm tr}\cdot\vec{B}$ is the row–column product, and the dot between two column vectors $\vec{A}\cdot\vec{B} = (\vec{A}^{\rm tr}\cdot g)\cdot\vec{B}$ is the product mediated by the metric. We shall avoid the last usage, however, limiting it to the 3–dimensional subspace

when a non–orthonormal basis is being used, so that we will write $\boldsymbol{v}\cdot\boldsymbol{a}$ instead of $\boldsymbol{v}^{c}\cdot\boldsymbol{a}$ for the product $v_k a^k = v^j \mathcal{G}_{jk} a^k$.

For brevity, since we shall deal only with a handful of bases, rather than indicating with $\vec{V}^{[e]}, \vec{V}^{[f]}$, etc., which basis a 4–vector is being represented in, we shall use one or more apexes, e.g. $\vec{V}, \vec{V}'$.

A.5 Lorentz transformations

In particular, a transformation between two frames both of which are using orthonormal bases is said to be a Lorentz transformation, which we will denote as L. In this case, the inverse satisfies

$$(L^{-1})^{\alpha}{}_{\beta} := \underline{e}^{(\alpha)}\cdot\underline{f}_{(\beta)} = \eta^{\alpha\alpha'}\eta_{\beta\beta'}\underline{e}_{(\alpha')}\cdot\underline{f}^{(\beta')}$$
$$= \begin{cases} (L^{\mathrm{tr}})^{\alpha}{}_{\beta}, \text{if } \alpha,\beta \in \{1,2,3\}, \text{ or if } \alpha=\beta=0 \\ -(L^{\mathrm{tr}})^{\alpha}{}_{\beta}, \text{if } \alpha \in \{1,2,3\} \text{ and } \beta = 0, \text{ or vice versa} \end{cases}. \tag{4}$$

Let $\boldsymbol{V}^{\mathrm{tr}} = -(L^0{}_1, L^0{}_2, L^0{}_3)$ the row 3–vector, $\boldsymbol{V}' = \{L^1{}_0, L^2{}_0, L^3{}_0\}$ the column 3–vector, and $\mathcal{L}$ the 3×3 spatial block of the transformation matrix, which thus has the block form

$$L = \begin{pmatrix} L^0{}_0 & -\boldsymbol{V}^{\mathrm{tr}} \\ \boldsymbol{V}' & \mathcal{L} \end{pmatrix}. \tag{5}$$

The conditions on the inverse Lorentz transformation can be written in the block matrix form

$$L^{-1} = \begin{pmatrix} L^0{}_0 & -\boldsymbol{V}'^{\mathrm{tr}} \\ \boldsymbol{V} & \mathcal{L}^{\mathrm{tr}} \end{pmatrix}. \tag{6}$$

By equalling $L^{-1}\cdot L$ and $L\cdot L^{-1}$ to the identity, we find the conditions:

$$[L^0{}_0]^2 = 1 + \boldsymbol{V}\cdot\boldsymbol{V} = 1 + \boldsymbol{V}'\cdot\boldsymbol{V}', \tag{7a}$$
$$\mathcal{L}\cdot\boldsymbol{V} + L^0{}_0\boldsymbol{V}' = \mathcal{L}^{\mathrm{tr}}\cdot\boldsymbol{V}' + L^0{}_0\boldsymbol{V} = 0, \tag{7b}$$
$$\mathcal{L}\cdot\mathcal{L}^{\mathrm{tr}} - \boldsymbol{V}'\boldsymbol{V}'^{\mathrm{tr}} = \mathcal{L}^{\mathrm{tr}}\cdot\mathcal{L} - \boldsymbol{V}\boldsymbol{V}^{\mathrm{tr}} = \mathbb{1}_3, \tag{7c}$$

or, in components

$$-L^{\alpha}{}_0 L^{\beta}{}_0 + \sum_j L^{\alpha}{}_j L^{\beta}{}_j = \eta^{\alpha\beta}, \tag{8a}$$
$$-L^0{}_{\alpha} L^0{}_{\beta} + \sum_j L^j{}_{\alpha} L^j{}_{\beta} = \eta_{\alpha\beta}. \tag{8b}$$

In Ref. [1], we discuss the classification of Lorentz transformations based on their eigenvalues, and on which axes, if any, they leave unchanged. In particular, here, two special cases are of interest: spatial rotations, which leave the time axis and a spatial axis unchanged, and boosts, which leave two spatial axes unchanged. Boosts are characterized by the property that they leave unchanged two spatial axes orthogonal to the relative velocity, which also implies that, if the velocity of the final frame relative to the initial frame is $\boldsymbol{v}$, the reciprocal

velocity $\boldsymbol{v}'$ is $-\boldsymbol{v}$. This latter property, however, is not unique to boosts, as it is shared with 4-screws [2], transformations consisting in a boost accompanied by a rotation about the axis of the boost. Spatial rotations, on the other hand, are characterized by a vector angle $\boldsymbol{\phi}$ encoding the direction of the rotation and the angle. Boosts are symmetric matrices, rotations are orthogonal matrices

$$B[\boldsymbol{v}] = \begin{pmatrix} \gamma & -\gamma \boldsymbol{v}^{\rm tr} \\ -\gamma \boldsymbol{v} & \mathbb{1}_3 + \frac{\gamma^2}{\gamma+1} \boldsymbol{v}\boldsymbol{v}^{\rm tr} \end{pmatrix}, \qquad R[\boldsymbol{\phi}] = \begin{pmatrix} 1 & \boldsymbol{0}^{\rm tr} \\ \boldsymbol{0} & \mathcal{R}[\boldsymbol{\phi}] \end{pmatrix}, \tag{9}$$

where $\mathcal{R}[\boldsymbol{\phi}] = \exp[\boldsymbol{\phi}\cdot\boldsymbol{\mathcal{J}}]$, with $\boldsymbol{\mathcal{J}}$ the vector having as components the 3×3 representations of the angular momentum

$$\mathcal{J}_1 = \begin{pmatrix} 0 & 0 & 0 \\ 0 & 0 & -1 \\ 0 & 1 & 0 \end{pmatrix}, \quad \mathcal{J}_2 = \begin{pmatrix} 0 & 0 & 1 \\ 0 & 0 & 0 \\ -1 & 0 & 0 \end{pmatrix}, \quad \mathcal{J}_3 = \begin{pmatrix} 0 & -1 & 0 \\ 1 & 0 & 0 \\ 0 & 0 & 0 \end{pmatrix}. \tag{10}$$

It is well known that a general proper orthochronous Lorentz transformation L can be decomposed uniquely as the product of a boost and a proper rotation:

$$L = R'[\boldsymbol{\phi}] \cdot B[\boldsymbol{v}] = B[\boldsymbol{v}^*] \cdot R[\boldsymbol{\phi}], \tag{11}$$

where $\boldsymbol{v}^* = \mathcal{R}[\boldsymbol{\phi}]\cdot\boldsymbol{v}$ is obtained by the rotation of the 3–vector $\boldsymbol{v}$ applying the spatial submatrix $\mathcal{R}$ of R.

The solutions to Eq. (11) are built as follows: For the decomposition $L = R'B$

- Take the first row of L in Eq. (5), input it as the first row of B in Eq. (9), and complete the entries of B. For orthochronous transformations $L^0{}_0 > 0$, hence this step is always possible. In components

$$B^\alpha{}_\beta[\boldsymbol{v}] = \eta^\alpha{}_\beta + \frac{(L^0{}_\alpha + \delta^0{}_\alpha)(L^0{}_\beta + \delta^0{}_\beta)}{L^0{}_0 + 1}. \tag{12}$$

- Consider the 3×3 matrix

$$\mathcal{R}' = \mathcal{L} + \frac{\boldsymbol{V}'\boldsymbol{V}^{\rm tr}}{L^0_0 + 1}. \tag{13}$$

  A straightforward verification using Eqs. (7) shows that it is an orthogonal matrix. It defines the left rotation R'. In components

$$\mathcal{R}'^j{}_k = L^j{}_k - \frac{L^j{}_0 L^0{}_k}{L^0{}_0 + 1}. \tag{14}$$

For the second decomposition, analogously, one takes the first column of L in Eq. (5), inputs it as the first column of $B[\boldsymbol{v}^*]$, and completes the entries according to Eq. (9). In components

$$B^\alpha{}_\beta[\boldsymbol{v}^*] = \eta^\alpha{}_\beta + \frac{(L^\alpha{}_0 + \delta^\alpha_0)(L^\beta{}_0 + \delta^\beta{}_0)}{L^0{}_0 + 1}. \tag{15}$$

The rotation matrix R is the same as R' in the previous decomposition.

A.5.1 Interpretation of the polar decompositions

The decomposition of the transformation between the frame $\mathfrak{S}$ and $\mathfrak{S}'$, $L = R'B$, means that the boost B gives the transformation from the frame $\mathfrak{S}$ to a frame $\mathfrak{S}'^*$ moving with velocity $\boldsymbol{v}$ relative to $\mathfrak{S}$ and hence at rest in $\mathfrak{S}'$, and relative to which $\mathfrak{S}$ moves with velocity $\boldsymbol{v}'^* = -\boldsymbol{v}$. The rotation R' represents a rotation in the boosted frame, hence the prime. The matrix R' transforms the representation of a 4–vector in $\mathfrak{S}'^*$ into its representation in $\mathfrak{S}'$, i.e. it describes how the axes of $\mathfrak{S}'^*$, obtained by a simple boost from $\mathfrak{S}$, are rotated relative to to the axes of the frame $\mathfrak{S}'$.

The alternative decomposition $L = BR$ has a different meaning. First one applies a rotation R in the frame $\mathfrak{S}$, thus representing a 4–vector in a frame $\mathfrak{S}^*$ at rest relative to $\mathfrak{S}$, but with the axes rotated. Then one applies a boost in this rotated frame, yielding the representation of the 4–vector in the final frame $\mathfrak{S}'$. The inverse matrix R^{-1} thus describes the rotation of $\mathfrak{S}^*$ relative to $\mathfrak{S}$. The fact that the frames are connected by a boost means that, since $\boldsymbol{v}^* = \mathcal{R}\boldsymbol{v}$ is the velocity of $\mathfrak{S}'$ relative to $\mathfrak{S}^*$, the velocity of $\mathfrak{S}^*$, and hence also of $\mathfrak{S}$, relative to $\mathfrak{S}'$ is $\boldsymbol{v}' = -\boldsymbol{v}^*$. For this reason, we denoted the first column of the Lorentz transformation (5) with the sign opposite to the first row: the column $\boldsymbol{V}' = \gamma'\boldsymbol{v}'$ yields directly the velocity of the original frame relative to the final frame.

Formally, the rotation matrices are the same $R = R'$. However, they represent different operations, hence we used a prime to distinguish them.

A.5.2 Minimal rotation

While the rotation $\mathcal{R}$ takes $\boldsymbol{v}$ to $\boldsymbol{v}^*$, in general, the rotation $\mathcal{R}$ is not minimal, i.e. it is not a rotation about the axis $\boldsymbol{v} \times \boldsymbol{v}^*$. In Minkowski space a rotation is no longer characterized by an axis and an angle, but rather by a plane and an angle. It seems appropriate to adapt to this characteristic when describing 3D rotations as well. In this case, by specifying any two unit vectors connected through $\hat{\boldsymbol{n}} = \mathcal{R}\hat{\boldsymbol{m}}$, and requiring that the rotation is in the plane determined by them, we arrive to the unique proper 3D–rotation satisfying these conditions,

$$\mathcal{R}(\hat{\boldsymbol{m}}, \hat{\boldsymbol{n}}) = \mathbb{1}_3 - \frac{1}{1 + \hat{\boldsymbol{m}}\cdot\hat{\boldsymbol{n}}}[\hat{\boldsymbol{m}} + \hat{\boldsymbol{n}}][\hat{\boldsymbol{m}}^{\rm tr} + \hat{\boldsymbol{n}}^{\rm tr}] + 2\hat{\boldsymbol{n}}\hat{\boldsymbol{m}}^{\rm tr} \ . \tag{16}$$

The rotation reduces to the identity when $\hat{\boldsymbol{m}} = \hat{\boldsymbol{n}}$, and it is, naturally, indefinite for $\hat{\boldsymbol{m}} = -\hat{\boldsymbol{n}}$, when a $\frac{0}{0}$ singularity appears.

In this case, indeed, writing $\hat{\boldsymbol{n}} = -\cos(\epsilon)\hat{\boldsymbol{m}} + \sin(\epsilon)[\cos(\phi_\epsilon)\hat{\boldsymbol{k}} + \sin(\phi_\epsilon)\hat{\boldsymbol{l}}]$, with $\hat{\boldsymbol{k}}, \hat{\boldsymbol{l}}, \hat{\boldsymbol{m}}$ an orthogonal triad, we see that there is no definite limit unless $\lim_{\epsilon\to 0}\phi_\epsilon$ exists.

The latter case means that $\hat{\boldsymbol{n}}$ tends to $-\hat{\boldsymbol{m}}$ on a fixed plane. Without loss of generality, let us say that $\lim_{\epsilon\to 0}\phi_\epsilon = 0$. Then taking the limit $\epsilon \to 0$, yields

$$\mathcal{R} \to \mathbb{1} - 2\hat{\boldsymbol{k}}\hat{\boldsymbol{k}}^{\rm tr} - 2\hat{\boldsymbol{m}}\hat{\boldsymbol{m}}^{\rm tr} \ , \tag{17}$$

which is a proper rotation of an angle π about the axis orthogonal to $\hat{\boldsymbol{m}}$ and $\hat{\boldsymbol{k}}$.

A.6 Thomas–Wigner's rotation and Stapp's formula

We apply the result about polar decomposition (11) to the combination of two boosts,

$$L = B[\boldsymbol{u'}]B[\boldsymbol{u}]. \tag{18}$$

We are using a prime on the second velocity because when applied to a vector representation $\vec{C}$, the first boost will transform a vector from its representation in a frame $\mathfrak{S}$ to its representation $\vec{C}'$ in a new frame $\mathfrak{S}'$, moving with velocity $\boldsymbol{u}$ relative to $\mathfrak{S}$. The second boost will represent $\vec{C}'$ in another frame $\mathfrak{S}''$, moving with velocity $\boldsymbol{u}'$ relative to $\mathfrak{S}'$. The product of two boosts, as described in my manuscript [1], individuates a special subset of Lorentz transformation, called planar transformations, which preserve the spatial axis orthogonal to the velocities.

It is convenient to work not with Newtonian velocities, but with the celerities $\boldsymbol{U} = \gamma_u \boldsymbol{u}$, $\boldsymbol{U}' = \gamma_{u'} \boldsymbol{u}'$. (It is not uncommon to read that $\gamma \boldsymbol{v}$ is the proper velocity. However, a proper quantity is one measured in a frame where a particle is momentarily at rest. Thus the proper velocity is always zero. We would like to use the term relativistic velocity for $\gamma \boldsymbol{v}$, but it is easily confused with relativistic speed meaning a speed close to that of light. To avoid this misnomer, we refer to $\gamma \boldsymbol{v}$ with the other attested name in the literature, celerity.) Then

$$\begin{aligned} L &= \begin{pmatrix} \gamma_{u'} & -\boldsymbol{U}'^{\mathrm{tr}} \\ -\boldsymbol{U}' & \mathbb{1}_3 + \frac{\boldsymbol{U}'\boldsymbol{U}'^{\mathrm{tr}}}{\gamma_{u'}+1} \end{pmatrix} \begin{pmatrix} \gamma_u & -\boldsymbol{U}^{\mathrm{tr}} \\ -\boldsymbol{U} & \mathbb{1}_3 + \frac{\boldsymbol{U}\boldsymbol{U}^{\mathrm{tr}}}{\gamma_u+1} \end{pmatrix} \\ &= \begin{pmatrix} \gamma_{u'}\gamma_u + \boldsymbol{U}'\cdot\boldsymbol{U} & -\boldsymbol{V}^{\mathrm{tr}} \\ +\boldsymbol{V}'' & \mathbb{1}_3 + \frac{\boldsymbol{U}'\boldsymbol{U}'^{\mathrm{tr}}}{\gamma_{u'}+1} + \frac{\boldsymbol{U}\boldsymbol{U}^{\mathrm{tr}}}{\gamma_u+1} + [1 + \frac{\boldsymbol{U}'\cdot\boldsymbol{U}}{(\gamma_{u'}+1)(\gamma_u+1)}]\boldsymbol{U}'\boldsymbol{U}^{\mathrm{tr}} \end{pmatrix}. \end{aligned} \tag{19}$$

Here, we put

$$\boldsymbol{V} = \boldsymbol{U'} + [\gamma_{u'} + \frac{\boldsymbol{U}'\cdot\boldsymbol{U}}{\gamma_u + 1}]\boldsymbol{U}, \tag{20a}$$

$$\boldsymbol{V}'' = -\boldsymbol{U} - [\gamma_u + \frac{\boldsymbol{U}'\cdot\boldsymbol{U}}{\gamma_{u'} + 1}]\boldsymbol{U}'. \tag{20b}$$

The vector $\boldsymbol{V}$ is but the celerity $\boldsymbol{U}'$ in the initial reference frame $\mathfrak{S}$, i.e. the celerity of $\mathfrak{S}''$ relative to $\mathfrak{S}$. Conversely, $\boldsymbol{V}''$ is the celerity of $\mathfrak{S}$ relative to $\mathfrak{S}''$.

It is impossible to have $\boldsymbol{V} = \boldsymbol{V}'' \neq \boldsymbol{0}$. Indeed, imposing $\boldsymbol{V} = \boldsymbol{V}''$ in Eqs. (20) we find

$$\frac{(\gamma_u + 1)(\gamma_{u'} + 1) + \boldsymbol{U}\cdot\boldsymbol{U}'}{\gamma_{u'} + 1}\boldsymbol{U}' = -\frac{(\gamma_u + 1)(\gamma_{u'} + 1) + \boldsymbol{U}\cdot\boldsymbol{U}'}{\gamma_u + 1}\boldsymbol{U}. \tag{21}$$

Since $(\gamma_u + 1)(\gamma_{u'} + 1) + \boldsymbol{U}\cdot\boldsymbol{U}' \geq \sqrt{(\gamma_u + 1)(\gamma_{u'} + 1)}[\sqrt{(\gamma_u + 1)(\gamma_{u'} + 1)} - \sqrt{(\gamma_u - 1)(\gamma_{u'} - 1)}] > 0$, it follows that Eq. (21) can hold only if $\boldsymbol{U} = -\boldsymbol{U}'$, which implies $\boldsymbol{V} = \boldsymbol{V}'' = \boldsymbol{0}$.

The common Lorentz factor of $\boldsymbol{V}$ and $\boldsymbol{V}''$ is

$$\gamma_v = \gamma_{u'}\gamma_u + \boldsymbol{U}'\cdot\boldsymbol{U}. \tag{22}$$

We shall use this relation to replace $\boldsymbol{U}'\cdot\boldsymbol{U}$ in terms of the Lorentz factors. In particular

$$\boldsymbol{V} = \boldsymbol{U'} + \frac{\gamma_{u'} + \gamma_v}{\gamma_u + 1}\boldsymbol{U}, \tag{23a}$$

$$\boldsymbol{V}'' = -\boldsymbol{U} - \frac{\gamma_u + \gamma_v}{\gamma_{u'} + 1}\boldsymbol{U}'. \tag{23b}$$

After replacing $\boldsymbol{U}$ and $\boldsymbol{U}'$ with $\boldsymbol{V}$ and $\boldsymbol{V}''$, we have

$$L = \begin{pmatrix} \gamma_v & -\boldsymbol{V}^{\rm tr} \\ \boldsymbol{V}'' & \mathbb{1}_3 - \frac{(\gamma_u+1)(\gamma_{u'}+1)}{(\gamma_v-1)(\gamma_u+\gamma_{u'}+\gamma_v+1)^2}[\boldsymbol{V}-\boldsymbol{V}''][\boldsymbol{V}^{\rm tr}-\boldsymbol{V}''^{\rm tr}] - \frac{\boldsymbol{V}''\boldsymbol{V}^{\rm tr}}{\gamma_v-1} \end{pmatrix}. \tag{24}$$

From the polar decomposition

$$L = R\cdot B[\boldsymbol{v}] = \begin{pmatrix} \gamma_v & -\boldsymbol{V}^{\rm tr} \\ -\mathcal{R}\cdot\boldsymbol{V} & \mathcal{R}\cdot(\mathbb{1}_3 + \frac{\boldsymbol{V}\boldsymbol{V}^{\rm tr}}{\gamma_v+1}) \end{pmatrix}, \tag{25}$$

we have, by comparison with Eq. (24),

$$\mathcal{R} = \mathbb{1}_3 - \frac{(\gamma_u+1)(\gamma_{u'}+1)(\gamma_v+1)}{(\gamma_u+\gamma_v+\gamma_{u'}+1)^2}[\hat{\boldsymbol{v}}-\hat{\boldsymbol{v}}''][\hat{\boldsymbol{v}}^{\rm tr}-\hat{\boldsymbol{v}}''^{\rm tr}] - 2\hat{\boldsymbol{v}}''\hat{\boldsymbol{v}}^{\rm tr} , \tag{26}$$

where we divided $\boldsymbol{V}$ and $\boldsymbol{V}''$ by their common norm $\sqrt{\gamma_v^2-1}$, introducing the corresponding unit vectors $\hat{\boldsymbol{v}}$ and $\hat{\boldsymbol{v}}''$.

The rotation matrix $\mathcal{R}$ is built combining only the identity and direct products of the vectors $\boldsymbol{V}$ and $\boldsymbol{V}''$ with themselves and with each other, hence, when applied to a vector $\hat{\boldsymbol{b}}$ orthogonal to both, it returns the same vector. Thus, $\mathcal{R}$ must describe a rotation around the axis orthogonal to both $\boldsymbol{V}$ and $\boldsymbol{V}''$, unless the velocities are antiparallel, in which case the rotation is the identity.

In other words, if a Lorentz transformation L is the product of two boosts, the rotation $\mathcal{R}$ defined in Eq. (14) is the minimal rotation taking the velocity $\boldsymbol{v}$ of the frame $\mathfrak{S}''$ relative to $\mathfrak{S}$ to the velocity $-\boldsymbol{v}''$, with $\boldsymbol{v}''$ the velocity of the frame $\mathfrak{S}$ relative to $\mathfrak{S}''$. Using the definition of the previous subsection A.5.2, the rotation is $\mathcal{R}(\hat{\boldsymbol{v}}, -\hat{\boldsymbol{v}}'')$. As we have established that it is impossible that $\boldsymbol{V} = \boldsymbol{V}'' \neq \boldsymbol{0}$, $\mathcal{R}$ is never indeterminate of the form $\frac{0}{0}$.

Since we have already deduced the axis (or better, the plane) of the rotation, in order to fully characterize the latter we need only provide the angle of rotation Θ. This is most easily achieved noting that since $\boldsymbol{V}'' = -\mathcal{R}\cdot\boldsymbol{V}$, applying $\mathcal{R}$ in Eq. (26) to $\boldsymbol{v}$, and imposing that it results in $-\boldsymbol{v}''$, one finds

$$1 - \frac{(\gamma_u+1)(\gamma_{u'}+1)(\gamma_v+1)}{(\gamma_u+\gamma_{u'}+\gamma_v+1)^2}(1-\hat{\boldsymbol{v}}\cdot\hat{\boldsymbol{v}}'') = 0 . \tag{27}$$

Thus, the angle Θ of the rotation $\mathcal{R}$ and the supplementary angle Φ between the velocity $\boldsymbol{v}$ of the frame $\mathfrak{S}''$ relative to $\mathfrak{S}$ and the velocity $\boldsymbol{v}''$ of the frame $\mathfrak{S}$ relative to $\mathfrak{S}''$ satisfy

$$1 + \cos\Theta = 1 - \cos\Phi = \frac{(\gamma_u+\gamma_{u'}+\gamma_v+1)^2}{(\gamma_u+1)(\gamma_{u'}+1)(\gamma_v+1)} . \tag{28}$$

This formula lacks determining the sense of the rotation, which we provide below. If we choose the orientation of the rotation axis $\hat{\boldsymbol{b}}$ to be such that $\boldsymbol{V}, -\boldsymbol{V}'' = \mathcal{R}\boldsymbol{V}, \hat{\boldsymbol{b}}$ form a right-handed triad,

$$\begin{aligned}\sin\Theta &= -\frac{(\boldsymbol{V}\times\boldsymbol{V}'')\cdot\hat{\boldsymbol{b}}}{|\boldsymbol{V}''||\boldsymbol{V}|} = -\frac{(\boldsymbol{V}\times\boldsymbol{V}'')\cdot\hat{\boldsymbol{b}}}{|\boldsymbol{V}|^2} = \left[\frac{\gamma_{u'}+\gamma_v}{\gamma_u+1}\frac{\gamma_u+\gamma_v}{\gamma_{u'}+1} - 1\right]\frac{(\boldsymbol{U}\times\boldsymbol{U}')\cdot\hat{\boldsymbol{b}}}{|\boldsymbol{V}|^2} \\ &= (\gamma_v - 1)\frac{\gamma_u+\gamma_v+\gamma_{u'}+1}{(\gamma_u+1)(\gamma_{u'}+1)}\frac{(\boldsymbol{U}\times\boldsymbol{V})\cdot\hat{\boldsymbol{b}}}{\gamma_v^2-1} \\ &= \frac{\gamma_u+\gamma_v+\gamma_{u'}+1}{(\gamma_u+1)(\gamma_v+1)(\gamma_{u'}+1)}|\boldsymbol{U}||\boldsymbol{V}|\sin\phi. \qquad (29)\end{aligned}$$

This is Stapp's formula [3]. Here we used $\boldsymbol{U}\times\boldsymbol{U}' = \boldsymbol{U}\times\boldsymbol{V}$, and defined ϕ as the smaller angle between $\boldsymbol{U}$ and $\boldsymbol{V}$. It is a quantity measurable in $\mathfrak{S}$. We may express $\gamma_{u'}$ as a function of quantities observable in the same frame $\mathfrak{S}$, using

$$\gamma_{u'} = \gamma_u\gamma_v - |\boldsymbol{U}||\boldsymbol{V}|\cos\phi. \qquad (30)$$

Finally, we find that the angle of the rotation satisfies [4]

$$\tan\frac{\Theta}{2} = \frac{\sin\Theta}{1+\cos\Theta} = \frac{|\boldsymbol{U}||\boldsymbol{V}|\sin\phi}{(1+\gamma_u)(1+\gamma_v) - |\boldsymbol{U}||\boldsymbol{V}|\cos\phi}, \qquad (31)$$

or better, in terms of hyperbolic functions, recalling that $|\boldsymbol{U}| = \sinh\theta_u$, $\gamma_u = \cosh\theta_u$, etc., with θ the relativistic rapidity,

$$\tan\frac{\Theta}{2} = \frac{\sin\phi}{\coth\frac{\theta_u}{2}\coth\frac{\theta_v}{2} - \cos\phi}. \qquad (32)$$

This equation allows to express the rotation in terms of quantities measured in $\mathfrak{S}$. It is a formula that may be useful whenever there is an actual reference frame $\mathfrak{S}'$, the velocity $\boldsymbol{u}$ of which $\mathfrak{S}$ will be able to measure, and it is known that the transformation from $\mathfrak{S}'$ and $\mathfrak{S}''$ is a pure boost.

A.6.1 Infinitesimal Thomas–Wigner rotation

A particularly interesting case, applicable to the problem under consideration of an accelerated reference frame, is when the second velocity $\boldsymbol{U}' \to \boldsymbol{\delta U}'$ is infinitesimal. Then the second boost takes the representation of a vector $\vec{C}'$ to the representation $\vec{C}''$ in an inertial frame infinitesimally close, the axes of which have been FW transported from the axes of $\mathfrak{S}'$, which is but the reference frame that, in the inertial frame $\mathfrak{S}$, is obtained from $\mathfrak{S}'$ by the velocity change $\boldsymbol{v} = \boldsymbol{u} + \boldsymbol{a}\delta t$ (in terms of Newtonian 3–velocities and acceleration). To first order in δt,

$$\gamma_{\delta u'} \simeq 1, \qquad \gamma_v \simeq \gamma_u + \gamma_u^3\boldsymbol{a}(t)\cdot\boldsymbol{u}(t)\delta t, \qquad (33)$$

thus the relativistic velocities appearing in the combined Lorentz transformation satisfy

$$\boldsymbol{V} = \boldsymbol{U} + \boldsymbol{\delta U} \simeq \boldsymbol{U} + \boldsymbol{\delta U}' + \frac{\gamma_u^3\boldsymbol{a}(t)\cdot\boldsymbol{u}(t)\delta t}{\gamma_u+1}\boldsymbol{U}, \qquad (34a)$$

$$\boldsymbol{V}'' \simeq -\boldsymbol{U} - \gamma_u\boldsymbol{\delta U}', \qquad (34b)$$

while the Newtonian velocities satisfy

$$\boldsymbol{v} = \boldsymbol{u} + \boldsymbol{\delta u} \simeq \boldsymbol{u} + \boldsymbol{\delta u}' - \frac{\gamma_u^2 \boldsymbol{a}(t)\cdot\boldsymbol{u}(t)\delta t}{\gamma_u + 1}\boldsymbol{u}, \tag{35a}$$

$$\boldsymbol{v}'' \simeq -\boldsymbol{u} - \boldsymbol{\delta u}' - \boldsymbol{a}(t)\cdot\boldsymbol{u}(t)\delta t\boldsymbol{u}, \tag{35b}$$

and the angle of rotation in Eq. (32) becomes

$$\delta\Theta \simeq (\gamma_u - 1)\delta\phi, \tag{36}$$

with $\delta\phi$ the infinitesimal angle between $\boldsymbol{u}$ and $\boldsymbol{v} = \boldsymbol{u} + \boldsymbol{a}\delta t$. The axis of the rotation is orthogonal to $\boldsymbol{u}$ and $\boldsymbol{u} + \boldsymbol{a}\delta t$, hence it is in the direction $\boldsymbol{u} \times \boldsymbol{a}$. In terms of boosts and rotation matrices, we have:

$$B[\boldsymbol{\delta v'}]B[\boldsymbol{u}] = R[\boldsymbol{\delta\Theta}]B[\boldsymbol{u} + \boldsymbol{\delta u}], \tag{37}$$

which we may rewrite as

$$R^{-1}[\boldsymbol{\delta\Theta}]B[\boldsymbol{\delta v'}] = B[\boldsymbol{u} + \boldsymbol{\delta u}]B[-\boldsymbol{u}], \tag{38}$$

Thus, we have rederived Thomas's correct (up to a sign) statement [5] that a boost $B[-\boldsymbol{u}]$ followed by $B[\boldsymbol{u}+\boldsymbol{\delta u}]$ is equivalent to an infinitesimal boost $B[\boldsymbol{\delta v'}]$ followed by an infinitesimal rotation $\boldsymbol{\delta\Theta} = -\frac{\gamma_u - 1}{u^2}\boldsymbol{u} \times \boldsymbol{\delta u}$.

A.7 The commutation hexagon

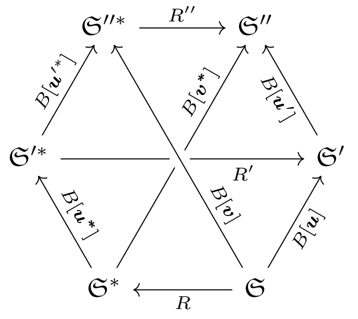

Figure 1: Diagrammatic representation of the commutation relations $B[\boldsymbol{u}']B[\boldsymbol{u}] = RB[\boldsymbol{v}] = B[\boldsymbol{v}^*]R$.

In Fig. 1, we represent diagrammatically the results of the previous section by means of a hexagon, the vertices of which represent different reference frames. Two frames connected by a horizontal line are at rest relative to each other, differing only by a given rotation R. The rotation is the same for all frames, i.e. it is represented by the same matrix. In the figure, $\boldsymbol{v}$ is the velocity of the double primed reference frames $\mathfrak{S}''$ and $\mathfrak{S}''^*$ relative to $\mathfrak{S}$, $\boldsymbol{u}'$ is the velocity of the double primed reference frames $\mathfrak{S}''$ and $\mathfrak{S}''^*$ relative to $\mathfrak{S}'$, and $\boldsymbol{u}$ is the

velocity of the primed reference frames $\mathfrak{S}'$ and $\mathfrak{S}'^*$ relative to $\mathfrak{S}$. The three velocities are thus related through the relativistic composition law. Starred velocities are relative to the starred frame $\mathfrak{S}^*$. The common rotation $R = R' = R'' = \left(\begin{smallmatrix} 1 & \mathbf{0}^{\mathrm{tr}} \\ \mathbf{0} & \mathcal{R} \end{smallmatrix}\right)$ is a rotation of an angle Θ (defined univocally in (32)) in the common plane of $\boldsymbol{u}, \boldsymbol{v}, \boldsymbol{u}'$. The starred 3–vectors $\boldsymbol{u}^*, \boldsymbol{v}^*$ are obtained from the respective unstarred vectors through the rotation $\mathcal{R}$ while the primed and starred vector $\boldsymbol{u}'^*$ is obtained by the inverse rotation $\mathcal{R}^{-1}\boldsymbol{u}'$. Thus the hexagon is determined by specifying any two velocities, e.g. $\boldsymbol{u}$ and $\boldsymbol{u}'$.

B The meaning of Thomas–Wigner rotation revealed through commutative diagrams

The pitfalls that lead to the error in Thomas precession could be avoided by using commutative diagrams for Lorentz transformations as a visual aid. It is well known that a Lorentz transformation from an inertial frame $\mathfrak{S}$ to an inertial frame $\mathfrak{S}'$ moving with velocity $\boldsymbol{v}$ relative to $\mathfrak{S}$ can be decomposed as the product of a boost and a rotation in two ways: $L = RB[\boldsymbol{v}]$ and $L = B[-\boldsymbol{v}']R$, with $\boldsymbol{v}'$ the velocity of $\mathfrak{S}$ relative to $\mathfrak{S}'$. We apply these decompositions to the series of Lorentz transformations from the lab frame to the comoving FW transported frames $\mathfrak{S}'_t$ the origins of which coincide with the position of an accelerated particle. Some care must be taken because dynamical quantities measured in the accelerated frame do not coincide with corresponding quantities measured in the instantaneous rest frame $\mathfrak{S}'_t$, since they involve incremental ratios of quantities measured in two different inertial frames $\mathfrak{S}'_t$ and $\mathfrak{S}'_{t+\delta t}$.

In the diagrams, boosts are indicated by dashed straight lines, rotations by dotted curved lines, and general Lorentz transformations by full curved lines. Two additional frames appear: a comoving frame, the axes of which rotate relative to the comoving FW frame, which we will call the comoving boosted frame $\mathfrak{S}'^*$; and a frame with the origin at rest in the lab, but rotating axes, which we will refer to as the rotating lab frame $\mathfrak{S}^*$. In the literature, the distinction between the FW frame and the rotating frame has been seldom recognized, with a few exceptions, as for instance the excellent book by Garg [6].

With reference to Fig. 2, let $\mathfrak{S}'_t$ a FW frame comoving with the accelerated particle. Let L_t the Lorentz transformations from the lab frame to the comoving FW frame at lab time t. Let $B[\boldsymbol{v}]$ the Lorentz boost from the lab frame to a frame centered at the particle and moving with velocity $\boldsymbol{v}$ relative to it, and let $\mathfrak{S}'^*_t$ the comoving frame defined by the boosts $B[\boldsymbol{v}_t]$ as the velocity of the particle varies in time. As the FW frame and the comoving boosted frame are at rest relative to each other, they are connected by a time–dependent rotation $R'_{t'}$, the finite Thomas–Wigner rotation, with t' their common proper time, a known function of t, so that $L_t = R'_{t'}B[\boldsymbol{v}_t]$. While there is no general analytic expression for $R'_{t'}$, its infinitesimal value is, as found by Thomas [5] (see the former section A.6.1 for a derivation with the correct sign)

$$\delta R'_{t'} = B[\boldsymbol{\delta v}'^*]B[\boldsymbol{v}_{t_0}]B[-\boldsymbol{v}_{t_0+\delta t}] = \mathbb{1}_4 + \frac{\gamma_t}{\gamma_t+1}(\boldsymbol{v}_t \times \boldsymbol{\delta v}'^*)\cdot\boldsymbol{J}, \qquad (39)$$

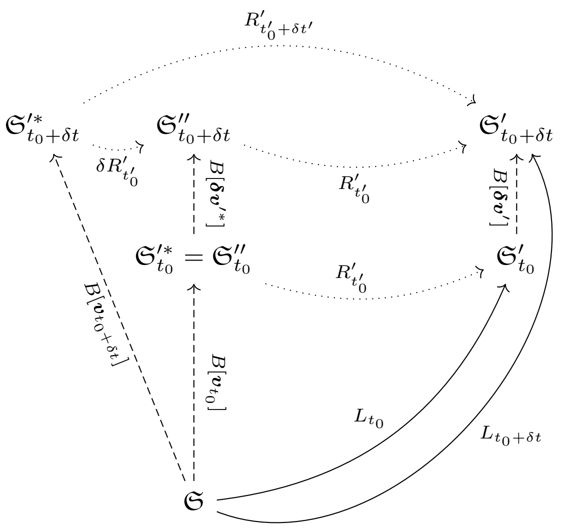

Figure 2: Commutative diagram for the polar decomposition of Lorentz transformations $L = RB[\boldsymbol{v}]$. We considered a generic time $t = t_0$ in the lab frame $\mathfrak{S}$, corresponding to a time t'_0 in any comoving frame, and an infinitesimal time interval δt, which is $\delta t'$ in any comoving frame. The primed and starred sequence of frames $\mathfrak{S}'^*_t$ is defined by applying pure Lorentz boosts to the lab frame. The primed sequence of frames $\mathfrak{S}'_t$ is obtained by FW transport. The double primed sequence of frames $\mathfrak{S}''_t$ is an auxiliary FW frame, the axes of which are rotated relative to $\mathfrak{S}'_t$ by the constant rotation $\mathcal{R}^{-1}_{t'_0}$.

with $\boldsymbol{J} = \left(\begin{smallmatrix} 1 & \boldsymbol{0}^{\rm tr} \\ \boldsymbol{0} & \boldsymbol{\mathcal{J}} \end{smallmatrix}\right)$ the 4×4 generators of spatial rotations and $\boldsymbol{\delta v}'^* = \boldsymbol{a}'^*_{t'} \delta t'$ the infinitesimal velocity increment measured in the boosted frame, $\boldsymbol{a}'^*$ being the proper acceleration measured in the comoving boosted frame.

From the commutative diagram Fig. 2, we infer that the rotation satisfies

$$R'_{t'+\delta t'} = R'_{t'} \delta R'_{t'} \ , \tag{40}$$

from which it follows immediately that the instantaneous angular velocity of the axes of $\mathfrak{S}'^*_t$ relative to the axes of $\mathfrak{S}'_t$ is

$$\boldsymbol{\omega}'_{t'} = \frac{\gamma_t}{\gamma_t + 1} \mathcal{R}_{t'} (\boldsymbol{v}_t \times \frac{\boldsymbol{\delta v}'^*}{\delta t'}) = -\frac{\gamma_t}{\gamma_t + 1} \boldsymbol{v}'_t \times \boldsymbol{a}'_t, \tag{41}$$

where we used the fact that $\boldsymbol{v}_t = -\boldsymbol{v}'^*_t$, the velocity of the frame $\mathfrak{S}$ relative to $\mathfrak{S}'^*$ is the opposite of $\boldsymbol{v}_t$, since the frames are connected by a boost, and the fact that applying $\mathcal{R}_{t'}$ to a vector of the boosted comoving frame $\mathfrak{S}'^*$ yields the corresponding vector in the comoving FW frame $\mathfrak{S}'$. The acceleration $\boldsymbol{a}'_t$ is thus the proper acceleration of the FW frame $\mathfrak{S}'$, while $\boldsymbol{v}'_t$ is the velocity of

$\mathfrak{S}$ relative to $\mathfrak{S}'$, in the sense that it is the velocity of the point of $\mathfrak{S}$ passing through the origin of $\mathfrak{S}'$ at time t'. Points at rest in an inertial frame appear to move, in an accelerated frame, with a position dependent velocity, similar to Hubble's law, in addition to the Coriolis velocity [7]. Thus, when stating the velocity of an inertial frame relative to an accelerated frame, one must specify which point of the inertial frame is being considered. The rotation of the axes of $\mathfrak{S}'_t$ relative to the axes of $\mathfrak{S}'^*_t$ is, instead

$$\boldsymbol{\omega}'^*_{t'} = \frac{\gamma_t}{\gamma_t + 1}\boldsymbol{v}'^*_t \times \boldsymbol{a}'^*_t. \tag{42}$$

In terms of quantities measured in the lab frame

$$\boldsymbol{\omega}'^*_{t'} = -\frac{\gamma_t^3}{\gamma_t + 1}\boldsymbol{v}_t \times \boldsymbol{a}_t. \tag{43}$$

Equation (43) is the source of a common misunderstanding on the Thomas–Wigner rotation: the rate on the lhs is observed in the boosted comoving frame of the particle, but the rhs is expressed as a function of quantities measured in the lab frame. This means merely that an inertial observer can predict what the accelerated observer will be measuring. However, a common mistake is to consider the lhs as if it was a quantity observed in the lab frame, and hence consider the rate in the lab frame, $\boldsymbol{\omega}'^*_t = \boldsymbol{\delta\Phi}/dt = -\frac{\gamma_t^2}{\gamma_t+1}\boldsymbol{v}_t \times \boldsymbol{a}_t$. This is the equation for Thomas precession, as most commonly derived.

We may proceed analogously for the alternative decomposition of Lorentz transformations, $L = BR$. In the commutative diagram 3, while the rotation is represented by the same matrix R as in the previous figure, it is a physically distinct operation, as it describes the rotation of the axes of $\mathfrak{S}^*_t$ relative to the axes of the lab frame $\mathfrak{S}$. Hence we are denoting it as R_t, with the time t measured in the lab frame and the rotating frame. From the commutative diagram Fig. 3, we infer that the rotation satisfies

$$R_{t+\delta t} = \delta R_t R_t\ . \tag{44}$$

The matrices R_t and $R'_{t'}$ coincide, for any $t' = \tau(t)$ simultaneous with the lab time t (according to the simultaneity of the lab frame: we recall that simultaneity in special relativity is not a reciprocal property). However, in this second decomposition the infinitesimal Thomas–Wigner rotation δR_t does not coincide with $\delta R'_{t'}$ in Eq. (39), but, as is clear comparing diagrams 2 and 3, it is

$$\delta R_t = B[-\boldsymbol{v}^*_{t_0+\delta t}]B[\boldsymbol{\delta v}']B[\boldsymbol{v}^*_{t_0}] = \mathbb{1}_4 + \frac{\gamma_t}{\gamma_t + 1}[(\mathcal{R}_t\boldsymbol{v}_t) \times \boldsymbol{\delta v}']\cdot\boldsymbol{J}, \tag{45}$$

Indeed, in order for the matrices R_t and $R'_{t'}$ to coincide identically in time, since they obey distinct differential equations $R'_{t'}\delta R' = R'_{t'+\delta t'}$ and $\delta R R_t = R_{t+\delta t}$, the increments δR and $\delta R'$ must be conjugated through R, $\delta R' = R^{-1}\delta R R$.

The axes of $\mathfrak{S}^*_t$ relative to the axes of $\mathfrak{S}$ transform with the inverse matrix, $\mathcal{R}_t^{-1}$. It follows immediately that the instantaneous angular velocity of the axes

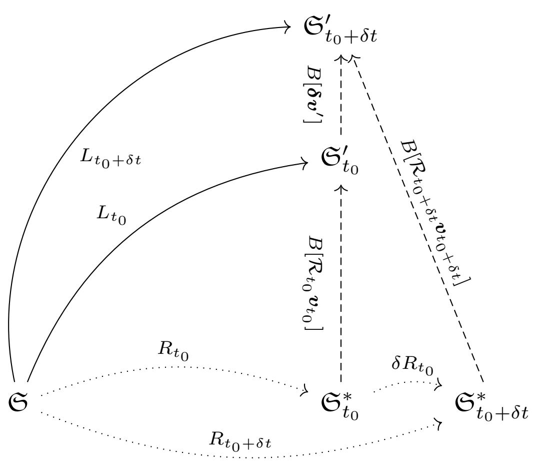

Figure 3: Commutative diagram for the alternative polar decomposition of Lorentz transformations $L = B[\mathcal{R}\boldsymbol{v}]R$. Here $\mathfrak{S}^*_t$ is a rotating frame with the origin at rest in the lab, defined as the frame obtained by applying the boosts $B[\boldsymbol{v}'_{t'}]$ to the comoving FW frame, with $\boldsymbol{v}'_{t'}$ being the velocity of the lab frame relative to the particle at time $t' = \tau(t)$.

of $\mathfrak{S}^*_t$ relative to the axes of $\mathfrak{S}$ is

$$\boldsymbol{\omega}_t = -\frac{\gamma_t}{\gamma_t + 1}\boldsymbol{v}_t \times \mathcal{R}_t^{-1}\frac{\boldsymbol{\delta v}'}{\delta t} = -\frac{\gamma_t^2}{\gamma_t + 1}\boldsymbol{v}_t \times \boldsymbol{a}_t \ . \tag{46}$$

Here, we used $\gamma_t \mathcal{R}_t^{-1}\frac{\boldsymbol{\delta v}'}{\delta t} = \mathcal{R}_t^{-1}\frac{\boldsymbol{\delta v}'}{\delta t'} = \boldsymbol{a}'^*_{t'} = \gamma_t^2 \boldsymbol{a}_t + \frac{\gamma_t^4}{\gamma_t+1}\boldsymbol{v}_t\cdot\boldsymbol{a}_t\boldsymbol{v}_t$, since $\boldsymbol{a}'^*_{t'}$ is the proper acceleration in the comoving boosted frame.

Finally, we have a quantity which is measured in the lab frame, given by Eq. (46): it is the angular velocity, relative to the lab frame, of a purely rotating frame, whose origin is at rest in the lab. The rotation (46) guarantees that the Lorentz transformation from the rotating frame to the FW accelerated frame is a Lorentz boost.

In summary, the Thomas–Wigner rotation describes the instantaneous rotation of a frame relative to another. It does not describe the rotation of a vector transported by an accelerated particle as seen in the lab frame, nor an additional Coriolis rotation seen by the accelerated particle. Rather, it describes the Coriolis rotation observed either in a rotating frame with the origin at rest relative to the lab frame, or in a frame comoving with the particle but the axes of which are not FW transported. Therefore, the Thomas–Wigner rotation rate while strictly related to the Thomas precession — defined as the rotation observed in the lab frame of the spin of a particle due only to relativistic kinematics effects — does not coincide with it, and thus can not possibly account for the energy levels of an atom at rest in the lab frame.

C The interplay of Thomas–Wigner rotation and Lorentz contraction in the spin dynamics

First of all, we establish a simple fact: pure Lorentz boosts imply a nonlinear rotation. Indeed, let us consider first a vector $\boldsymbol{P}$ associated to a particle initially at rest in the lab frame. If the particle is boosted by a velocity $\boldsymbol{\Delta v}$, the vector $\boldsymbol{P}$ will contract in the direction of the velocity, as illustrated in the figure 4. Therefore, the vector $\boldsymbol{P}$ will rotate by an angle $\Delta\theta$, depending on its orientation relative to the velocity boost.

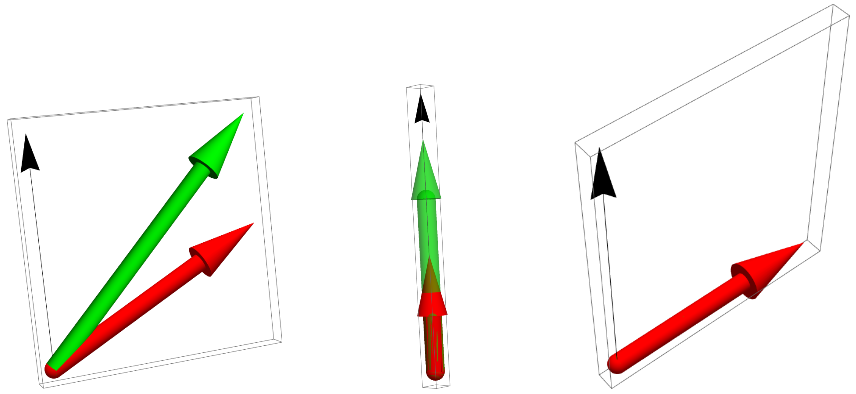

Figure 4: A 3D vector which is represented as the green arrow in a reference frame, is represented by the red arrow in a new reference frame boosted with velocity $\boldsymbol{v}$ (black arrow) with respect to the former frame. In general, the red vector will be rotated relative to the green vector, because of Lorentz contraction. Here, we chose a Lorentz factor $\gamma = 2$ for illustrative purposes.

The angle $\Delta\theta$ is zero when $\boldsymbol{P}$ is normal, parallel, or antiparallel to the direction of the boost, and it reaches a maximum when the angle α between $\boldsymbol{P}$ and $\boldsymbol{\Delta v}$ satisfies $\cos(2\alpha) = \frac{\gamma_{\Delta v}-1}{\gamma_{\Delta v}+1}$, which yields $\alpha \to 0, \pi$ in the ultra–relativistic limit, and $\alpha \simeq \pi/4$ in the non–relativistic limit (Fig. 5).

This rotation combines with the rotation of the vector due to external influences, and also with the Thomas–Wigner rotation. Precisely, we have shown that the Lorentz transformation from the lab frame to the FW rest frame of the particle is $L_{\mathrm{FW}}(t) = R_{\mathrm{TW}}(t)B[\boldsymbol{v}_t]$. Hence, since in the rest frame the spin is represented as the 4–vector: $\{0, \boldsymbol{S}'\}$, the corresponding representation in the lab frame is

$$\begin{pmatrix} S^0(t) \\ \boldsymbol{S}_t \end{pmatrix} = B[-\boldsymbol{v}_t]R^{\mathrm{tr}}_{\mathrm{TW}}(t)\begin{pmatrix} 0 \\ \boldsymbol{S}'_t \end{pmatrix}, \tag{47}$$

where we used the fact that the inverse of a Lorentz boost $B[\boldsymbol{v}]$ is $B^{-1}[\boldsymbol{v}] = B[-\boldsymbol{v}]$, and the inverse of a rotation is $R^{-1} = R^{\mathrm{tr}}$.

The relevant spatial part is

$$\boldsymbol{S}_t = \mathcal{R}^{\mathrm{tr}}_{\mathrm{TW}}(t)\boldsymbol{S}'_t + (\gamma_t - 1)\hat{\boldsymbol{v}}_t\hat{\boldsymbol{v}}^{\mathrm{tr}}_t\mathcal{R}^{\mathrm{tr}}_{\mathrm{TW}}(t)\boldsymbol{S}'_t. \tag{48}$$

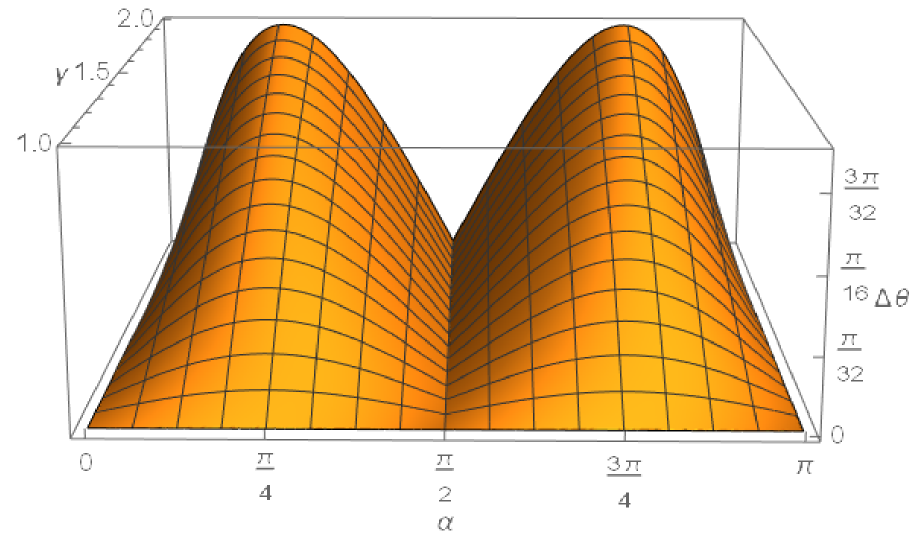

Figure 5: The angle of rotation $\Delta\theta$ of a vector $\boldsymbol{P}$ due to Lorentz contraction as a function of the angle α that the vector makes with the velocity of the boost, for several values of γ.

Here, we used Eqs. (9). The second term on the rhs describes the dilation of the vector $\boldsymbol{S}$ along the velocity axis.

For the simultaneity–corrected vector $\boldsymbol{T}_t$, instead

$$\boldsymbol{T}_t = \mathcal{R}_{\mathrm{TW}}^{\mathrm{tr}}(t)\boldsymbol{S}'_t + (\gamma_t^{-1} - 1)\hat{\boldsymbol{v}}_t\hat{\boldsymbol{v}}_t^{\mathrm{tr}}\mathcal{R}_{\mathrm{TW}}^{\mathrm{tr}}(t)\boldsymbol{S}'_t. \tag{49}$$

The second term on the rhs describes the contraction of the vector $\boldsymbol{T}$ along the velocity axis.

Let us assume temporarily that we could neglect the effects of Lorentz contraction/dilation. Then

$$\boldsymbol{S}_t \simeq \boldsymbol{T}_t \simeq \mathcal{R}_{\mathrm{TW}}^{\mathrm{tr}}(t)\boldsymbol{S}'_t. \tag{50}$$

In the comoving FW frame, the spin $\boldsymbol{S}'$ makes a Larmor precession due to the electromagnetic field acting on the particle therein,

$$\frac{d}{dt'}\boldsymbol{S}'_{t'} = \boldsymbol{\omega}'_{\mathrm{L}}(t) \times \boldsymbol{S}'_{t'} = \Omega'_{\mathrm{L}}(t)\boldsymbol{S}'_{t'}, \tag{51}$$

where Ω'_{L} is the antisymmetric matrix associated to the vector $\boldsymbol{\omega}'_{\mathrm{L}}$. In the lab frame, therefore, neglecting consistently, within the approximation of the present paragraph, the difference between the factor $\gamma_t = \frac{dt'}{dt}$ and 1,

$$\begin{aligned}\frac{d}{dt}\boldsymbol{S}_t &\simeq \left[\mathcal{R}_{\mathrm{TW}}^{\mathrm{tr}}(t)\Omega'_{\mathrm{L}}\mathcal{R}_{\mathrm{TW}}(t) + \Omega_{\mathrm{TW}}(t)\right]\mathcal{R}_{\mathrm{TW}}^{\mathrm{tr}}(t)\boldsymbol{S}'_t \\ &= \left[\Omega_{\mathrm{L}}(t) + \Omega_{\mathrm{TW}}(t)\right]\boldsymbol{S}_t = \left[\boldsymbol{\omega}_{\mathrm{L}}(t) + \boldsymbol{\omega}_{\mathrm{TW}}(t)\right] \times \boldsymbol{S}_t.\end{aligned} \tag{52}$$

Here, we introduced the Larmor frequency in the lab frame, $\Omega_{\mathrm{L}}(t)=\mathcal{R}^{\mathrm{tr}}_{\mathrm{TW}}(t)\Omega'_{\mathrm{L}}\mathcal{R}_{\mathrm{TW}}(t)$, $\boldsymbol{\omega}_{\mathrm{L}}(t)=\mathcal{R}^{\mathrm{tr}}_{\mathrm{TW}}(t)\boldsymbol{\omega}'_{\mathrm{L}}(t)$ and the Thomas–Wigner rotation frequency, defined by $\frac{d}{dt}\mathcal{R}^{\mathrm{tr}}_{\mathrm{TW}}(t)=\Omega_{\mathrm{TW}}(t)\mathcal{R}^{\mathrm{tr}}_{\mathrm{TW}}(t)$. From Eq. (36), with $\boldsymbol{v}_t\to\boldsymbol{u}$, we find

$$\begin{aligned}\boldsymbol{\omega}_{\mathrm{TW}}(t) &:= -\frac{\delta\Theta}{\delta t}\frac{\boldsymbol{v}_t\times\boldsymbol{a}_t}{|\boldsymbol{v}_t\times\boldsymbol{a}_t|} = -(\gamma_t-1)\frac{\boldsymbol{v}_t\times(\boldsymbol{v}_t+\boldsymbol{a}_t\delta t)}{|\boldsymbol{v}_t\times(\boldsymbol{v}_t+\boldsymbol{a}_t\delta t)|}\frac{\delta\phi}{\delta t}\\ &\simeq -(\gamma_t-1)\frac{\boldsymbol{v}_t\times\boldsymbol{a}_t\delta t}{\boldsymbol{v}_t^2\sin(\delta\phi)}\frac{\delta\phi}{\delta t}\simeq -(\gamma_t-1)\frac{\boldsymbol{v}_t\times\boldsymbol{a}_t}{\boldsymbol{v}_t^2}. \end{aligned} \tag{53}$$

Due to the presence of γ_t-1, the additional Thomas–Wigner rotation, however, is of the same order of the contributions due to the Lorentz contraction/dilation which were neglected. Thus, the approximation is not consistent. Furthermore, we wish to know the dynamics of the spin also in the relativistic regime.

Therefore, we need the full Eq. (48) or Eq. (49), which do not conserve the norm of the spin 3–vector. In these equations, the effects of the Thomas–Wigner rotation $\mathcal{R}_{\mathrm{TW}}$ and of the Lorentz contraction/dilation $(\gamma_t^{\pm1}-1)\hat{\boldsymbol{v}}_t\hat{\boldsymbol{v}}_t^{\mathrm{tr}}$ get twined.

Indeed, let $\mathcal{L}_t^+=\mathbb{1}_3+(\gamma_t-1)\hat{\boldsymbol{v}}_t\hat{\boldsymbol{v}}_t^{\mathrm{tr}}$ the 3×3 matrix describing Lorentz dilation, $\mathcal{L}_t^-=\mathbb{1}_3+(\gamma_t^{-1}-1)\hat{\boldsymbol{v}}_t\hat{\boldsymbol{v}}_t^{\mathrm{tr}}$ the 3×3 matrix, inverse of $\mathcal{L}^+$, describing Lorentz contraction, and $\boldsymbol{P}^+=\boldsymbol{S}$, $\boldsymbol{P}^-=\boldsymbol{T}$. Upon differentiation of Eq. (48) and Eq. (49), there are terms which involve $\mathcal{L}^\pm$, $\mathcal{R}_{\mathrm{TW}}$, and their derivatives:

$$\frac{d}{dt}\boldsymbol{P}_t^\pm=[\frac{d}{dt}\mathcal{L}_t^\pm]\mathcal{L}_t^\mp\boldsymbol{P}_t^\pm+\mathcal{L}_t^\pm\Omega_{\mathrm{TW}}(t)\mathcal{L}_t^\mp\boldsymbol{P}_t^\pm+\Omega_{\mathrm{L}}^\pm(t)\boldsymbol{P}_t^\pm, \tag{54}$$

with the Larmor matrix in the lab frame $\Omega_{\mathrm{L}}^\pm=\gamma\mathcal{L}^\pm\mathcal{R}^{\mathrm{tr}}_{\mathrm{TW}}\Omega'_{\mathrm{L}}\mathcal{R}_{\mathrm{TW}}\mathcal{L}^\mp$, no longer a skew–symmetric matrix as Ω'_{L}.

Thus, one cannot say "this term is due to the Lorentz contraction, this other term is due to the Thomas–Wigner rotation." In order to extract a rotation, one should use the technique illustrated in the Methods section of the main manuscript.

References

[1] Di Lorenzo, A. A new eigenvalue-based classification of Lorentz transformations. To be submitted.

[2] Synge, J. L. *Relativity: The Special Theory* (North-Holland Publishing Company, 1956).

[3] Stapp, H. P. Relativistic theory of polarization phenomena. *Phys. Rev.* **103**, 425 (1956).

[4] Ritus, V. I. Transformations of the inhomogeneous Lorentz group and the relativistic kinematics of polarized states. *Zh. Eksp. Teor. Fiz.* **40**, 352 (1961). [Sov. Phys. JETP **13** 240 (1961)].

[5] Thomas, L. H. I. the kinematics of an electron with an axis. *Philos. Mag. Ser. 7* **3**, 1 (1927).

[6] Garg, A. *Classical Electromagnetism in a Nutshell* (Princeton University Press, 2012).

[7] Di Lorenzo, A. Spacetime accounting for non inertial observers. In preparation.
