# Supplementary material for: Nonlinear effects in Thomas precession due to the interplay of Lorentz contraction and Thomas–Wigner rotation
Source: Sci Rep. 2022 Oct 6;12:16757. doi: 10.1038/s41598-022-20942-w (PMC9537424; doi:10.1038/s41598-022-20942-w)
Supplement: Supplementary file 2 — Supplementary Information 2. [file 41598_2022_20942_MOESM2_ESM.docx]

**Nonlinear effects in Thomas precession due to the interplay of Lorentz contraction and Thomas-Wigner rotation**

**Video S1:** The four relevant reference frames, in the exemplary case of a particle in uniform circular motion. The solid axes attached to the red dot individuate the lab frame **S**. The solid axes attached to the black dot individuate the Fermi—Walker comoving frame **S’**. The dotted axes attached to the red dot individuate the purely rotating auxiliary frame **S^*^**. Finally, the dotted axes attached to the black dot individuate the boosted comoving frame **S’^*^.** The rotation rate **ω** of the axes of **S^*^** measured in the lab frame is commonly mistaken in the literature for the precession rate of the spin of the particle in the lab frame, which instead is shown in videos 2 and 3. The rotation rate **ω**’**^*^**of the axes of **S’** relative to **S’^*^** differs from the rotation rate **ω** by the Lorentz factor γ, due to time dilation.
A Lorentz factor γ=5/3 was used.

**Video S2a:** The arrow in red shows the spin $\boldsymbol{T}$ defined according to the relativity of simultaneity for a particle making a uniform circular motion.
**Video S2b:**The arrow in green shows the spin direction $\hat{\boldsymbol{T}}$.
Because of Lorentz contraction, the spin points mostly in the radial direction. The nonlinear terms in the angular velocity make the spin whip quickly in and out.
